# Quantitative Preclinical Imaging as a Metrological Framework: Reproducibility, Validation, and Translational Maturity

**DOI:** 10.3390/jimaging12060242

**Published:** 2026-05-29

**Authors:** Nicolò Lauciello, Giorgio Russo, Alessandro Stefano

**Affiliations:** 1 Department of Earth and Marine Sciences, University of Palermo, Via Archirafi 22, 90123 Palermo, Italy; nicolo.lauciello@unipa.it; 2 Institute of Bioimaging and Complex Biological Systems, National Research Council, 90015 Cefalù, Italy; alessandro.stefano@cnr.it; 3 National Laboratory of South, National Institute for Nuclear Physics (LNS-INFN), 95123 Catania, Italy

**Keywords:** preclinical imaging, quantitative imaging biomarkers, standardization and harmonization, nuclear imaging, computed tomography, magnetic resonance imaging

## Abstract

Quantitative preclinical imaging enables non-invasive characterization of physiological, molecular, and functional processes providing measurable biomarkers for longitudinal and translational studies. This review systematically analyzes 60 studies published between 2015 and 2025, covering major imaging modalities including Positron emission tomography (PET), Single-Photon Emission Computed Tomography (SPECT), Magnetic resonance imaging (MRI), Computed Tomography (CT), optical imaging, and hybrid systems across murine and zebrafish models. We examine methodological frameworks for parameter extraction, reproducibility, and validation against biological reference standards, evaluating each modality through a cross-cutting analytical framework that distinguishes technical, biological, and computational sources of quantitative variance and identifies the current metrological maturity of harmonization infrastructure across platforms. Key comparative findings indicate that variability sources can be broadly categorized into technical (instrumentation, reconstruction, calibration) and biological (physiological heterogeneity, model-specific factors), with their interaction governing overall measurement uncertainty. Emerging computational approaches, including parametric modeling and artificial intelligence–assisted pipelines, show potential in reducing variance and improving parameter stability, although they introduce additional dependencies requiring validation. Collectively, this review frames quantitative preclinical imaging as a metrological discipline, emphasizing that reproducibility, bias control, and cross-modality harmonization are critical for generating robust and translationally relevant imaging biomarkers.

## 1. Introduction

The transition from qualitative to quantitative imaging in preclinical research represents one of the most consequential methodological developments in biomedical science, with roots in quantitative nuclear medicine extending back several decades and accelerating substantially with advances in computational reconstruction and hybrid imaging over the past two decades. Qualitative interpretation relies on visual assessment of image contrast, whereas quantitative frameworks extract standardized numerical parameters that are biologically interpretable, reproducible, and scalable across experimental platforms. When imaging-derived metrics inform therapeutic response assessment, biomarker validation, or cross-species extrapolation, their metrological properties—reproducibility, systematic bias, and parameter identifiability—become as scientifically decisive as the physical sensitivity of the acquisition system itself [1,2]. While quantitative imaging principles have been extensively formalized in clinical settings, this review focuses explicitly on preclinical imaging, where methodological implications differ fundamentally in terms of spatial scale, acquisition geometry, biological variability, and validation opportunities. In preclinical research, quantitative imaging operates within controlled experimental environments that enable invasive biological validation, prospective protocol manipulation, and longitudinal within-subject designs that are not available in routine clinical practice.

For the purposes of this review, preclinical imaging is defined as the application of non-invasive or minimally invasive imaging techniques to experimental biological models in research rather than direct clinical care context, encompassing small mammals (primarily mice and rats), rabbit models, and zebrafish (*Danio rerio*). Zebrafish were included because of their established and growing role in quantitative preclinical imaging research, particularly owing to their optical transparency during larval stages, rapid development, genetic tractability, and suitability for high-throughput phenotyping, which have motivated dedicated quantitative imaging method development across multiple modalities. Other vertebrate models (e.g., birds and reptiles) were not included because they have not generated a comparably established body of quantitative imaging methodology within the scope of the reviewed literature.

Preclinical imaging encompasses a heterogeneous spectrum of modalities, each offering distinct quantitative capabilities and limitations. PET and SPECT provide highly sensitive molecular and functional information through radiotracer concentration estimates, yet their quantitative reliability is critically dependent on acquisition settings, reconstruction algorithms, attenuation and scatter correction strategies, and system calibration [3,4]. Magnetic Resonance Imaging (MRI) offers multiparametric tissue characterization—including diffusion, perfusion, relaxometry, and metabolic profiling—but its reproducibility across platforms is sensitive to protocol heterogeneity and implementation variability [5,6,7]. Computed Tomography (CT) and micro-CT provide attenuation-based structural metrics traceable to physical units, enabling reproducible assessment of skeletal microarchitecture, lung morphology, and tumor volumes when workflows are harmonized [8,9]. Optical and photoacoustic modalities provide high-throughput molecular sensitivity and compatibility with longitudinal designs, but remain challenged by photon scattering, depth-dependent attenuation, and the ill-posed nature of optical inverse problems [10,11,12,13]. Despite these advances, a key limitation constrains the translational value of preclinical quantitative imaging: the lack of shared metrological frameworks enabling comparison across platforms, laboratories, and biological models. Inter-laboratory variability, hardware-dependent bias, heterogeneous pipelines, and inconsistent validation practices undermine reproducibility [3,4,14,15]. While prior reviews have largely focused on individual imaging modalities or specific technical aspects, this review adopts a unified metrological framework that distinguishes technical, biological, and computational sources of quantitative variability, enabling structured cross-modality evaluation of reproducibility, quantitative performance, and translational readiness. Importantly, unlike clinical imaging, preclinical harmonization is further constrained by small-animal physiology, partial-volume effects, limited signal-to-noise regimes, and species-specific biological validation requirements. Although standardized acquisition and quality-control frameworks are relatively mature in PET imaging [1,2,4,14], comparable harmonization infrastructures remain limited across MRI, CT, optical, and photoacoustic modalities.

This review intentionally focuses on PET, SPECT, MRI, CT/micro-CT, optical, and photoacoustic imaging modalities. Ultrasonography—including echocardiography, high-frequency ultrasound, 3D ultrasound, and ultrasound elastography—and classical fluoroscopy-based techniques (X-ray, angiography) were not included. This exclusion reflects a methodological rather than scientific prioritisation: these modalities have substantial translational potential and established preclinical applications, but their quantitative frameworks, harmonization challenges, and metrological infrastructure differ substantially from those of the modalities reviewed here and would require dedicated analytical treatment. We acknowledge this as a limitation and encourage future reviews to address ultrasound- and fluoroscopy-based quantitative imaging within comparable metrological frameworks.

Emerging computational approaches—encompassing parametric mapping, AI-assisted reconstruction, and standardized post-processing pipelines—offer promising tools to mitigate these limitations. However, they simultaneously introduce additional layers of algorithmic dependency that themselves require transparent validation before quantitative outputs can be considered biologically interpretable [3,4,13,16].

Emphasis is placed on identifying where harmonization infrastructure is sufficiently mature to support robust biomarker generation, and where methodological gaps persist that limit cross-platform comparability and translational applicability. By combining methodological analysis with advances in computational and multimodal imaging, this review aims to guide the development of reproducible and translationally relevant imaging biomarkers [5,17,18,19,20]. Consequently, this review adopts a preclinical perspective, using selected concepts from clinical quantitative imaging only to the extent that they contribute to metrological interpretation and translational relevance.

## 2. Materials and Methods

### 2.1. Literature Searching Strategy

This review was designed as a structured narrative review with systematic elements, aimed at enabling critical methodological comparison and metrological synthesis across preclinical imaging modalities rather than exhaustive evidence aggregation. The search strategy was designed to capture studies addressing quantitative imaging methodologies, reproducibility, validation, and translational applicability across multiple preclinical imaging modalities. Bibliographic research was performed in the main scientific databases: PubMed, Scopus, and Web of Science, including studies published between 2015 and 2026 (see Table 1). The temporal boundary was selected to capture the contemporary landscape of quantitative preclinical imaging, a period characterized by substantial advances in iterative reconstruction, AI-assisted analysis, and hybrid platform development. Foundational contributions predating this period are acknowledged in the Introduction and modality-specific sections where historically significant methodological milestones are relevant to understanding current practices. The search was restricted to articles published in English. This limitation was applied to ensure consistent interpretation of technical terminology, quantitative definitions, and validation methodologies, which are essential for the metrological analysis conducted in this review. The study identification and selection workflow was documented following Preferred Reporting Items for Systematic Reviews and Meta-Analyses (PRISMA) inspired reporting principles to enhance transparency and reproducibility, while being adapted to the conceptual scope of an integrative methodological review [21]. Search strings were constructed by combining terms describing quantitative imaging and parameter extraction with descriptors of experimental models and imaging modalities: (“quantitative imaging” OR “parametric mapping “ OR “functional imaging” OR “reproducibility” OR “validation” OR “parameter extraction”) AND (“preclinical” OR “small animal” OR “murine” OR “zebrafish”) AND (MRI” OR “diffusion MRI” OR “DCE-MRI” OR “relaxometry” OR “IVIM” OR “diffusion kurtosis” OR “PET” OR ‘’micro-CT/CT’’ OR “micro-PET” OR “functional PET” OR “dynamic PET” OR “SPECT” OR “micro-SPECT” OR “functional SPECT” OR “optical imaging” OR “fluorescence imaging” OR “bioluminescence imaging” OR “photoacoustic imaging” OR “optoacoustic imaging”). Relevant studies were also identified through manual screening of references from selected articles, ensuring broad coverage of methodological, reproducibility, and translational aspects. In addition, search terms were iteratively refined to capture modality-specific terminology and variations in the definition of quantitative imaging across disciplines. Studies based exclusively on qualitative image interpretation were excluded, as they do not provide quantitative metrics amenable to reproducibility analysis, variance decomposition, or metrological benchmarking, which constitute the primary focus of this review.

### 2.2. Inclusion and Exclusion Criteria

To ensure consistency and relevance, strict inclusion and exclusion criteria were defined. Studies that met the following criteria were considered eligible:

- Implementation of quantitative imaging approaches with explicitly defined measurable endpoints, including model-based parameters (e.g., kinetic, diffusion, or perfusion models), calibrated physical measurements (e.g., attenuation coefficients or activity concentration), or imaging biomarkers supported by biological or histological validation.
- Application within preclinical experimental models, including small animal studies or ex vivo imaging frameworks relevant to translational research.
- Explicit consideration of methodological aspects related to quantification, such as reproducibility, standardization, calibration, or validation of imaging-derived parameters.

The following were excluded:

- Absence of clearly defined quantitative endpoints, including studies limited to qualitative image interpretation or descriptive analysis without measurable parameters.
- Lack of methodological description regarding the derivation, validation, or reproducibility of imaging-derived metrics.
- Studies focused exclusively on clinical imaging without a preclinical component.

This selection allowed us to focus our analysis on studies that provide concrete evidence of quantitative preclinical imaging applications, while ensuring the possibility of comparing methods and results between different studies. For the purposes of this review, quantitative imaging was defined as the extraction of measurable parameters from imaging data that are either physically calibrated, mathematically modeled, or biologically validated, enabling reproducible and comparable assessment across experimental conditions. Eligible publication types included peer-reviewed original research articles and review articles. Conference abstracts, editorials, commentaries, letters, and other non-peer-reviewed publications were excluded due to insufficient methodological detail and lack of reproducibility or validation data. Grey literature sources, including theses, technical reports, preprints, and unpublished studies, were not systematically included. While such sources may offer contextual insights, they were excluded to ensure consistency, traceability, and quality control across quantitative assessments. Formal PRISMA components specific to systematic reviews, such as protocol registration, quantitative risk-of-bias assessment, and meta-analytic synthesis, were not implemented, as they were not aligned with the objectives of this metrological and cross-modality methodological analysis.

### 2.3. Classification of Studies

Selected studies were grouped according to three dimensions:

- Biological model: murine, zebrafish, rabbit.
- Imaging modality: CT/micro- CT, MRI, PET, SPECT, optical imaging, photoacoustic imaging.
- Type of quantitative approach: diffusion-based metrics, perfusion modelling, relaxometry, spectroscopy, AI-assisted mapping, or multimodal integration.

This framework allows organization of the Results section into coherent thematic categories, enabling comparison across imaging modalities, experimental models, and methodological strategies, while highlighting reproducibility challenges and emerging trends. The three-dimensional classification was conceived as an organizational and analytical framework to support structured comparison across heterogeneous studies. Formal statistical aggregation or meta-analytic synthesis was not performed, as variability in models, quantitative endpoints, and validation strategies would preclude meaningful pooled analysis. This approach is consistent with established recommendations for evidence synthesis under high methodological heterogeneity, where structured narrative frameworks are considered more appropriate than quantitative meta-analysis [22,23].

## 3. Results

### 3.1. Study Selection

**Figure 1:**
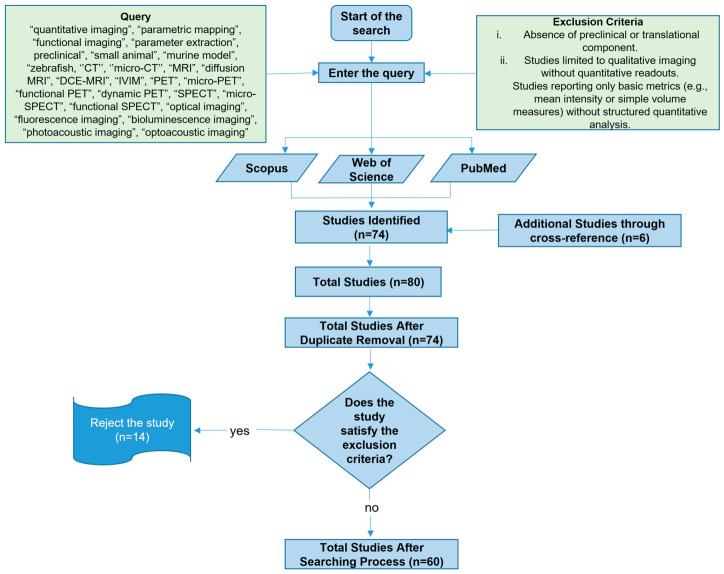
PRISMA-style flow diagram summarizing the literature search, screening, and study selection process adopted in this review; arrows indicate the sequential workflow of the study. Different colors are used to distinguish between queries, criteria, and the subsequent procedural steps.

The results are organized according to imaging modality and quantitative framework, enabling a structured comparison across nuclear, magnetic resonance, optical, and hybrid techniques in preclinical research. Within each category, studies are further stratified by biological model and application domain to facilitate cross-modality evaluation of quantitative performance and translational relevance. The selected articles are summarized in structured tables and critically discussed with respect to study objectives, acquisition and reconstruction protocols, modeling strategies, and primary quantitative endpoints. Emphasis is placed on harmonization procedures, reproducibility metrics, bias-variance considerations, and methodological constraints affecting parameter robustness and inter-study comparability. The study selection process is outlined in Figure 1. An initial total of 80 records was identified through structured database searches (PubMed, Scopus, and Web of Science) and complementary cross-reference screening. After removal of duplicates, 74 unique records remained. Following title and abstract screening, 14 studies were excluded for not meeting the predefined inclusion criteria. This final dataset provides a balanced and methodologically diverse representation of quantitative preclinical imaging approaches across modalities and biological models.

### 3.2. Overview of Biological Models and Imaging Modalities

**Figure 2:**
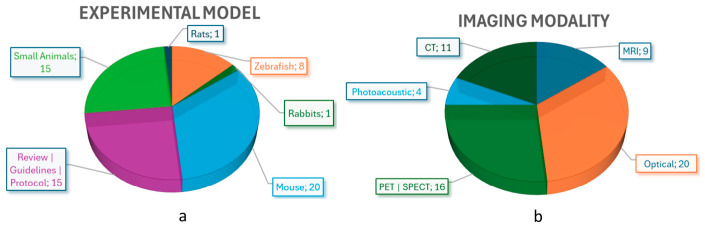
Pie charts illustrating the distribution of included studies by (**a**) experimental model and (**b**) imaging modality. Studies employing multimodal approaches are categorized by their primary imaging modality for the purposes of this distribution.

The included studies were categorized according to imaging modality—CT, MRI, PET, SPECT, photoacoustic imaging, optical imaging, and explicitly multimodal approaches—and by experimental model (e.g., murine, rabbit, and zebrafish). The term “small animal” generally refers to rodent models (primarily mice and rats), although other species, such as rabbits or zebrafish, may also be encompassed depending on the imaging modality and application. This distribution is illustrated in Figure 2, with panel a showing the distribution by experimental model and panel b showing the proportion of studies by imaging modality. The charts highlight the methodological diversity of contemporary preclinical imaging research.

### 3.3. Variance, Bias, and Cross-Modality Harmonization in Preclinical Imaging

**Figure 3:**
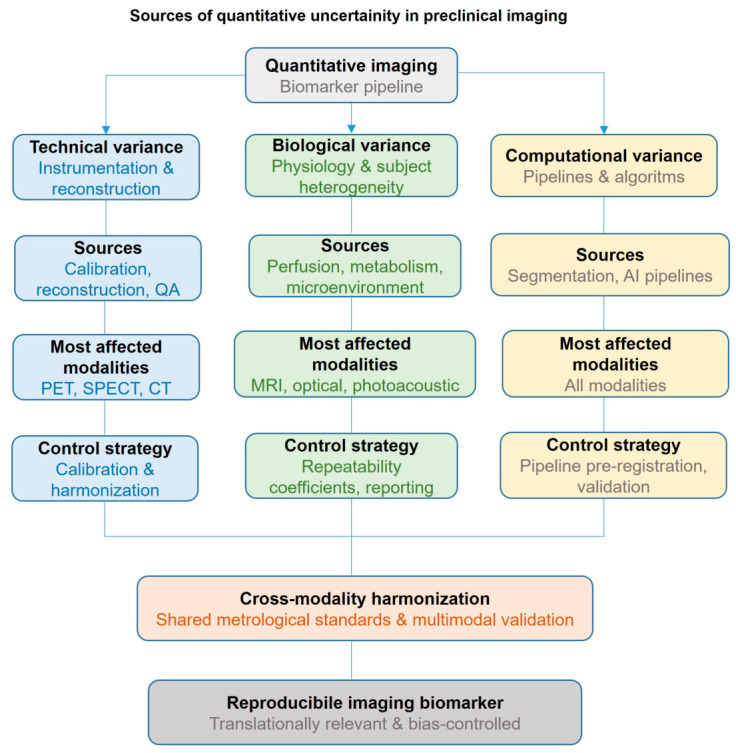
Conceptual framework of variance sources in quantitative preclinical imaging pipelines; arrows indicate the directional flow of the analysis. Different colors are used to distinguish among error types, examples, and conclusions.

Quantitative robustness in preclinical imaging does not derive from instrumental sensitivity alone, but from the controlled management of error sources distributed across the entire imaging pipeline. A conceptually useful framework distinguishes three principal categories of variability, as seen in Figure 3: technical variance, arising from instrumentation, calibration, and reconstruction; biological variance, reflecting genuine physiological heterogeneity across subjects and timepoints; and computational variance, introduced by post-processing algorithms, segmentation strategies, and analytical pipelines. In formal terms, the total variance of a quantitative imaging measurement can be approximated as:

$${Var}_{total}={Var}_{tech}+{Var}_{bio}+{Var}_{comp}$$

where the individual terms represent technical, biological, and computational sources of variability, respectively. This additive formulation assumes approximate independence between variance components and is conceptually consistent with variance component analysis frameworks commonly used in quantitative imaging and measurement science. Although a full empirical decomposition across modalities is beyond the scope of this review, this formalization provides a rigorous interpretive basis for the comparative framework discussed in the following sections. While the distinction between technical and biological sources of variance is well established, the present framework explicitly incorporates computational variance arising from reconstruction algorithms, kinetic modeling, and AI-assisted analysis, enabling a more systematic cross-modality interpretation of quantitative performance in preclinical imaging. Disentangling these components is a prerequisite for meaningful cross-study and cross-platform comparison, and failure to do so systematically undermines the metrological credibility of derived imaging biomarkers. Cross-species transfer of quantitative imaging protocols requires a distinction between acquisition parameter adjustment and full re-validation. Adjustments in field of view, resolution, or coil configuration accommodate differences in scale and do not invalidate the underlying quantitative framework. Re-validation is required only when biological differences alter the relationship between the imaging signal and the measured parameter, as is typically the case for functional and model-based biomarkers, but not for structural or directly physical metrics. Formal metrological tools—including repeatability coefficients, reproducibility coefficients, and intraclass correlation analysis—provide the statistical infrastructure necessary to quantify and transparently report these variance components, enabling objective cross-platform comparison of quantitative biomarker performance [24,25]. It is important to distinguish between physical calibration standards and biological reference standards. For structural and densitometric quantification—such as bone mineral density from micro-CT or chemical shift referencing in MRS—physical phantom standards (e.g., hydroxyapatite phantoms and tetramethylsilane (TMS) as the 0 ppm reference in ^1^H-MRS) are sufficient for metrological traceability and do not require biological validation. Biological reference standards are instead required for functional and molecular imaging biomarkers, where the signal depends on underlying biological processes. In this context, repeatability reflects measurement variability under identical acquisition conditions, whereas reproducibility captures variability introduced across systems, operators, or centers; the intraclass correlation coefficient (ICC) estimates the proportion of total variance attributable to true biological differences relative to measurement-related variability. The modality-specific manifestations of each variance category are discussed in detail in the sections that follow; the present framework is intended to provide the interpretive lens through which those sections should be read. The metrological maturity of harmonization infrastructure varies substantially across modalities, and this asymmetry has direct consequences for translational applicability. Metrological maturity reflects the extent to which a modality satisfies criteria including standardized protocols, validated calibration, demonstrated reproducibility across centres, robust parameter estimation, and established biological validation—operationalized in full in Supplementary Table S1. This continuum of maturity provides the basis for the comparative assessment presented in Table 2.

Cross-modality comparison highlights that imaging techniques differ not only in absolute quantification capability, but also in how technical, biological, and computational sources of variance propagate through the measurement pipeline, thereby affecting reproducibility and translational robustness. In general, PET and SPECT benefit from the most mature harmonization infrastructures and comparatively controlled technical variance, whereas MRI reproducibility remains more sensitive to protocol heterogeneity and vendor-dependent implementation. CT and micro-CT provide physically traceable quantitative metrics with relatively low computational variance, although without fully harmonized multicenter accreditation frameworks. By contrast, optical and photoacoustic imaging are more strongly constrained by computational and depth-dependent technical variability associated with inverse reconstruction problems and tissue-dependent signal attenuation. This modality-specific variance profile provides the interpretive basis for the comparative metrological assessment presented in Table 2.

Nuclear imaging—particularly PET—currently benefits from the most developed accreditation ecosystem, supported by multicenter standardization initiatives and international guidelines from the European Association of Nuclear Medicine (EANM) and European Society for Molecular Imaging (ESMI) [14,16]. MRI harmonization is progressing through consensus recommendations from the International Society for Magnetic Resonance in Medicine (ISMRM), but remains constrained by vendor heterogeneity and the absence of universally adopted phantom standards [6,26]. It should also be noted that the substantial consolidation of the preclinical imaging instrumentation market may reduce some sources of technical variability within single-vendor environments, while simultaneously increasing dependence on proprietary reconstruction and calibration procedures that are not independently auditable. This represents an important step towards harmonization, but multicenter accreditation remains incomplete with respect to PET/SPECT. For CT, calibration practices (e.g., Hounsfield Unit consistency, phantom-based quality control, and protocol standardization) remain challenging due to the absence of a single, globally unified and widely harmonized accreditation framework comparable to those established in nuclear medicine. For optical and photoacoustic imaging, published work has proposed several standardization and calibration approaches (e.g., fluorescence signal normalization, reconstruction benchmarking, and workflow harmonization), although these remain largely methodological, with limited multicenter validation and no widely adopted accreditation framework comparable to those in more mature quantitative imaging domains [27]. Multimodal integration strategies—such as constraining optical reconstructions with anatomical MRI priors, or cross-validating optical readouts against nuclear biodistribution data—can partially mitigate modality-specific bias and stabilize ill-posed inverse problems, but require explicit co-validation against shared biological reference standards to be metrologically credible [5,20]. These considerations define a practical framework for the design of quantitative preclinical imaging studies. Controlling technical variance requires prospective protocol harmonization, phantom-based calibration, and reconstruction standardization before data acquisition begins. Characterizing biological variance requires adequate cohort sizing, longitudinal within-subject designs where feasible, and explicit reporting of repeatability and reproducibility coefficients alongside primary quantitative endpoints. Mitigating computational variance requires pipeline pre-registration, version control of analytical software, and independent validation of AI-assisted components. Achieving cross-modality comparability, finally, requires not only technical alignment but biological co-validation—ensuring that parameters derived from different modalities are anchored to shared ground-truth reference standards. When these principles are systematically implemented, quantitative preclinical imaging can fulfil its promise as a reproducible, biologically interpretable, and translationally relevant measurement science [24,25,28].

Table 2 summarizes the comparative quantitative assessment across all six metrological dimensions, with scores assigned according to predefined criteria fully defined and operationalized in Supplementary Table S1; the modality-specific strategies, reproducibility data, and validation approaches underlying each assessment are discussed in the sections that follow. Quantitative performance scores represent a structured qualitative synthesis of the reviewed evidence based on the predefined criteria reported in Supplementary Table S1 and were assigned through interpretive comparative assessment rather than formal quantitative meta-analysis.

### 3.4. Quantitative CT in Preclinical Imaging

CT and micro-CT provide quantitative imaging based on X-ray attenuation, enabling direct measurement of tissue density through Hounsfield Units. In preclinical systems, micro-CT achieves spatial resolutions of ~5–50 μm, while in vivo micro-CT for small animals typically achieves ~0.1–0.3 mm spatial resolution depending on system configuration and dose constraints. These modalities support quantitative measurements such as bone mineral density (via hydroxyapatite calibration), tissue density, and volumetric segmentation with high geometric fidelity. Quantitative outputs are strongly influenced by acquisition parameters, reconstruction algorithms, and segmentation strategies, although attenuation values retain physical traceability when properly calibrated using reference phantoms such as hydroxyapatite standards. Table 3 summarises representative quantitative applications across skeletal, pulmonary, tumor, and vascular domains.

The studies included in this section are summarized in Table 3, which were selected to represent the breadth of quantitative CT and micro-CT applications reviewed, covering skeletal, pulmonary, vascular, and tumor domains, as well as methodological frameworks addressing calibration, reconstruction, and AI-assisted analysis. Selection was guided by the predefined inclusion criteria described in Section 2.2, with priority given to studies that explicitly report quantitative endpoints, reproducibility metrics, or validation data. This table is intended as a structured interpretive summary rather than an exhaustive catalogue, and the absence of a study does not imply exclusion from the qualitative synthesis provided in the narrative. Among the available preclinical imaging modalities, CT and micro-CT occupy a methodologically distinct position: their quantitative output is grounded in physical attenuation measurements expressed as Hounsfield Units, providing a more direct physically grounded quantitative framework than modalities relying on kinetic modeling or optical inverse problems. This physical traceability, however, does not eliminate the need for rigorous protocol harmonization—reproducibility of CT-derived metrics remains critically dependent on acquisition settings, reconstruction algorithms, and segmentation strategies, as reviewed comprehensively by [8]. Compared with nuclear imaging, quantitative CT benefits from a fundamentally different metrological basis: Hounsfield Unit-based measurements are grounded in physical attenuation coefficients that do not require kinetic modeling or radiopharmaceutical standardization, conferring direct physical traceability for structural and densitometric endpoints. However, CT currently lacks the internationally coordinated accreditation and harmonization frameworks that support PET/SPECT reproducibility across centers. Compared with MRI, CT offers more direct physical interpretability but cannot match the multiparametric depth of tissue characterization—including diffusion, perfusion, and relaxometry—achievable through advanced MRI protocols. The principal strength of quantitative CT and micro-CT therefore lies in reproducible structural and densitometric phenotyping within harmonized acquisition workflows, rather than in biologically comprehensive multiparametric characterization or cross-platform biological comparability, where complementary modalities currently benefit from more mature validation infrastructures. Quantitative robustness is most rigorously established for skeletal applications. Protocol-dependent sensitivity of these metrics to voxel size and segmentation method represents a recognized source of systematic variance that must be prospectively controlled [29]. Reproducibility of densitometric and biomechanical descriptors—including bone mineral density, cortical thickness, and trabecular architecture metrics such as bone volume fraction BV/TV and trabecular thickness Tb.Th—has been formally validated with repeatability and reproducibility coefficients reported across longitudinal in vivo protocols [30]. Extending beyond skeletal assessment, micro-CT has demonstrated comparable quantitative utility in pulmonary research. Standardized longitudinal workflows enable reproducible extraction of total lung volume, aerated lung fraction, and mean lung density across disease progression models [31]. Fully automated deep learning pipelines have further advanced quantitative consistency in this domain, delivering high-accuracy densitometric assessment of pulmonary fibrosis with reduced operator dependency and validated correlation to histopathological endpoints [32,33,34]. Reproducibility benchmarking has been further supported by the availability of annotated whole-body micro-CT databases in tumor-bearing murine models, enabling systematic evaluation of segmentation consistency and inter-observer variability across automated and manual pipelines [35]. Quantitative radiomics analyses extending structural assessment into texture-based descriptors have also been validated, although cross-platform reproducibility of radiomic features remains dependent on harmonized acquisition and reconstruction workflows—a limitation that persists across cone-beam and micro-CT systems [9]. Non-invasive skeletal muscle quantification represents a further emerging application, with morphometric descriptors including muscle volume and cross-sectional area demonstrating methodological feasibility, pending formal reproducibility validation [36]. Beyond structural and densitometric applications, contrast-enhanced micro-CT with nanoparticle agents extends quantitative capability toward vascular architecture and perfusion mapping, enabling semi-quantitative assessment of vascular volume fraction and contrast distribution in vivo [37]. Collectively, these findings position quantitative CT and micro-CT as a modality with high absolute quantification capability and increasingly mature AI-assisted analysis pipelines, but with harmonization infrastructure that remains less developed than nuclear imaging—particularly for cross-platform radiomics and vascular applications. Controlling beam hardening, scatter, and partial volume effects through standardized reconstruction, combined with prospective dose optimization in longitudinal survival studies, represents the critical prerequisite for extracting reproducible, biologically interpretable structural biomarkers within integrated preclinical imaging frameworks, consistent with the variance control principles outlined in Section 3.3.

### 3.5. Quantitative MRI in Preclinical Models

Quantitative MRI enables multiparametric assessment of tissue structure, diffusion, perfusion, relaxation, susceptibility, and metabolism based on proton spin behavior in magnetic fields. In preclinical systems, spatial resolution typically ranges from ~50–200 μm in vivo depending on field strength, coil configuration, and acquisition strategy. Quantitative accuracy is strongly dependent on sequence design, reconstruction methods, and vendor-specific implementation, which introduce protocol-dependent variability across studies. Table 4 summarises representative quantitative MRI applications across diffusion, perfusion, relaxometry, susceptibility, and spectroscopic domains.

The studies included in this section are summarized in Table 4, which provides a structured overview of quantitative MRI applications in preclinical models, detailing the primary quantitative focus, biological model, key quantitative outcome, and level of validated quantitative performance for each included study. Quantitative MRI encompasses a substantially broader methodological landscape than can be comprehensively addressed within a single section. The techniques discussed here reflect those most extensively represented in the reviewed preclinical literature and therefore do not constitute an exhaustive catalogue of quantitative MRI capabilities. Important quantitative domains not specifically covered include MR thermometry, pH-sensitive CEST imaging, cardiac functional quantification (e.g., ejection fraction and myocardial strain), and direct volumetric analysis. These omissions reflect the scope of the reviewed literature rather than their scientific relevance. Among quantitative MRI techniques, reproducibility is most rigorously established for diffusion-based metrics, whereas perfusion and relaxometry parameters remain more vulnerable to protocol heterogeneity and vendor-dependent implementation variability. Moreover, vendor-specific MRI implementations represent a recognised and practically consequential source of systematic variance in quantitative preclinical studies. Differences in gradient performance, RF pulse design, sequence implementation, and default reconstruction settings introduce cross-vendor discrepancies in derived parameters—including ADC, T\u2081, and T\u2082 values—that are not attributable to genuine biological differences. The ISMRM guidelines for diffusion MRI explicitly identify gradient non-linearity, eddy current compensation, and b-value accuracy as key implementation-dependent factors requiring prospective calibration [6]. In the absence of vendor-independent phantom standards and cross-system calibration protocols, these implementation discrepancies constitute a persistent barrier to multi-center and multi-system comparability in quantitative MRI, highlighting the continuing need for vendor-independent harmonization frameworks in quantitative MRI. DWI and its primary derived parameter—ADC—represent the most extensively validated quantitative MRI biomarkers in preclinical research. Structured ISMRM recommendations further provide guidance on acquisition parameters, gradient calibration, motion management, and modeling strategies to ensure quantitative robustness across preclinical systems [6]. DWI has additionally been validated on preclinical PET/MRI hybrid platforms, confirming compatibility with multiparametric workflows [38]. Beyond conventional ADC, advanced diffusion approaches—including Intravoxel Incoherent Motion (IVIM) and Diffusion Kurtosis Imaging (DKI)—further separate perfusion and diffusion contributions while capturing non-Gaussian diffusion behavior, enhancing microstructural characterization and enabling correlation with histological endpoints such as hypoxia biomarkers [39,40]. Building on diffusion analysis, Dynamic Contrast-Enhanced MRI (DCE-MRI) enables quantitative assessment of tissue perfusion and vascular permeability through pharmacokinetic modeling. Accurate estimation of arterial input function (AIF) is central to parameter extraction, and optimised automated detection strategies reduce operator dependency while improving reproducibility of parameters such as volume transfer constant (K^trans^) and extravascular extracellular volume fraction (v_e) [41]. Emerging multiparametric approaches further advance this landscape: 3D MR Fingerprinting integrates simultaneous T_1_/T_2_ mapping with dynamic contrast acquisition in a single scan, providing comprehensive tissue characterization while reducing acquisition time [20]. Quantitative relaxometry provides direct measurement of tissue T_1_ and T_2_/T_2_* relaxation times, sensitive to microenvironmental changes including edema, fibrosis, and tumor progression. A critical methodological distinction should nevertheless be emphasised: many preclinical studies reporting T_2_ measurements quantify an apparent or effective T_2_ value rather than the true spin-spin relaxation time, owing to sequence-dependent dephasing contributions, susceptibility effects, diffusion weighting, and practical limitations in small-animal acquisitions. Consequently, T_2_-derived parameters should be interpreted cautiously unless acquisition protocols explicitly demonstrate robust spin-echo–based quantification under controlled conditions. These approaches are increasingly applied for therapy monitoring and longitudinal studies, with between-session variability formally quantified even under controlled experimental conditions—underscoring the necessity of explicitly separating biological fluctuation from instrumental noise [42]. Complementary techniques further expand the quantitative MRI toolkit: Quantitative Susceptibility Mapping (QSM) enables assessment of tissue magnetic susceptibility for investigations of iron deposition, hemorrhage, and calcification [43], while Proton Magnetic Resonance Spectroscopy (^1^H-MRS) provides metabolic profiling through quantification of absolute or relative metabolite concentrations, increasingly combined with relaxometry in multiparametric protocols [44]. Across the quantitative MRI techniques reviewed, reproducibility maturity varies substantially. Diffusion-based metrics—particularly ADC—represent the most extensively validated quantitative MRI parameters, supported by ISMRM consensus recommendations and multicenter benchmarking data. Advanced diffusion approaches (IVIM, DKI) extend quantitative capability but lack equivalent multicenter reproducibility evidence, with most studies reporting single-center validation. DCE-MRI parameters (K\u1D40\u02B3\u1D43\u207F\u02E2, v\u2091) achieve moderate reproducibility when AIF estimation is optimised but remain sensitive to modeling assumptions and protocol heterogeneity. Relaxometry parameters (T\u2081, T\u2082, T\u2082*) demonstrate measurable between-session variability even under controlled experimental conditions, reflecting both biological and instrumental contributions that require prospective separation. Susceptibility mapping and spectroscopy remain comparatively early-stage in preclinical validation, with limited reproducibility data reported. This hierarchy of reproducibility maturity has direct implications for study design: diffusion-based biomarkers currently offer the most reliable quantitative MRI endpoints, while perfusion and relaxometry parameters require more rigorous protocol harmonization before cross-platform deployment. Collectively, these findings position quantitative MRI as the modality with the highest multiparametric depth among those reviewed, capable of simultaneously characterizing diffusion, perfusion, relaxation, susceptibility, and metabolic properties within integrated preclinical workflows. This richness, however, is counterbalanced by pronounced sensitivity to protocol heterogeneity and the absence of universally adopted cross-vendor phantom calibration standards— a gap that constrains harmonization maturity relative to nuclear imaging and represents the primary target for methodological development, consistent with the framework outlined in Section 3.3.

### 3.6. Quantitative Preclinical Nuclear Imaging: PET and SPECT

PET and SPECT provide quantitative molecular imaging based on radiotracer distribution and gamma photon detection, enabling absolute or semi-quantitative assessment of biological processes. In preclinical systems, PET typically achieves spatial resolutions of ~1–2 mm, while SPECT ranges from ~0.5–1.5 mm depending on collimation and system design. Quantitative accuracy is influenced by reconstruction algorithms, attenuation and scatter correction, and calibration procedures, although tracer uptake measurements retain a strong physical basis when properly standardized. Table 5 summarises representative applications of quantitative PET and SPECT in preclinical molecular imaging.

The studies included in this section are summarized in Table 5, which provides a structured overview of quantitative nuclear medicine applications in preclinical models, detailing the primary quantitative focus, biological model, key quantitative outcome, and level of validated quantitative performance for each included study. Among the modalities reviewed, nuclear imaging—and particularly PET—currently exhibits one of the most mature metrological infrastructures, primarily due to the availability of traceable absolute quantification procedures, standardized calibration workflows, and comparatively advanced harmonization initiatives. Quantitative PET readouts most commonly begin with the Standardized Uptake Value (SUV), which normalizes tissue radioactivity to injected dose and body metrics. SUV reproducibility, however, is critically dependent on harmonized acquisition and analysis protocols. Multicentric preclinical studies have shown that uncontrolled inter-system SUV variability may exceed 20%, whereas standardization of injection procedures, scan timing, attenuation correction, reconstruction settings, and scanner dose-calibrator cross-calibration substantially improves quantitative consistency across platforms [17]. These sources of SUV variance—spanning injection-related, reconstruction-related, and calibration-related components—further illustrate the importance of harmonized workflows within the variance decomposition framework outlined in Section 3.3. Importantly, PET’s comparatively advanced metrological readiness reflects primarily the maturity of its harmonization ecosystem and calibration infrastructure, rather than the absence of quantitative limitations. Static SUV remains a simplified surrogate of underlying tracer kinetics and is sensitive to multiple physiological and methodological confounders, including body composition, blood glucose levels, uptake timing, and reconstruction settings. Dynamic PET and kinetic modeling provide more physiologically meaningful parameter estimation, but simultaneously increase sensitivity to arterial input function uncertainty, statistical noise, and parameter identifiability constraints—particularly in small-animal models characterized by limited blood volume and reduced counting statistics [45,46]. In addition, partial volume effects at preclinical spatial scales represent a major source of systematic bias requiring dedicated correction strategies. Therefore, the higher metrological maturity attributed to PET in Table 2 should be interpreted as reflecting the relative advancement of standardization and calibration infrastructures, rather than universal quantitative superiority. In addition, independent radiopharmaceutical and biochemical assays remain necessary to validate that imaging-derived quantitative parameters accurately reflect underlying biological processes. Dynamic PET extends quantitative capability beyond static SUV by enabling extraction of Time-Activity Curves (TACs) and estimation of kinetic rate constants through compartmental and graphical modeling frameworks, providing deeper physiological insight into tracer distribution and metabolism [47]. Moreover, AI-assisted arterial input function estimation has further demonstrated the potential to enhance kinetic parameter precision and reduce operator dependency in small-animal PET models [46]. Translational evaluations comparing dedicated preclinical PET with clinical total-body PET/CT systems have shown encouraging quantitative concordance, reinforcing the translational scalability of rigorous preclinical PET methodologies—while simultaneously highlighting scale-dependent challenges including partial volume effects at near-organism spatial resolution [3]. Quantitative PET imaging has further demonstrated the capacity to capture therapy-induced biological alterations in experimental tumor models, reinforcing its role as a mechanistic indicator of treatment response and translational biomarker discovery [48]. Beyond oncological applications, PET has been applied for quantitative molecular imaging of neuroinflammation in rodent models, enabling assessment of microglial activation and inflammatory biomarkers with histological validation [49], as well as for biodistribution analysis of novel radiotracers [50]. Cross-species feasibility has additionally been demonstrated in adult zebrafish, where measurable radiotracer uptake and longitudinal imaging capability extend quantitative PET methodologies to small non-mammalian vertebrate models—a development that requires explicit metrological re-validation at each new biological scale [51]. Turning to SPECT, advances in multi-pinhole collimation, iterative reconstruction with resolution recovery, attenuation and scatter correction, and system-response modeling have substantially narrowed the quantitative gap with PET, although quantitative SPECT remains intrinsically constrained by lower sensitivity and count statistics, particularly in dynamic acquisition settings. Absolute activity quantification becomes achievable when datasets are corrected for physical degrading factors through CT-based attenuation correction and calibrated iterative reconstruction. Quantitative accuracy further depends on phantom-derived calibration factors, scatter correction quality, and precise incorporation of system-response modeling within the reconstruction algorithm [52]. Contemporary multi-isotope small-animal SPECT systems have been validated for simultaneous radionuclide imaging with preserved quantitative integrity, expanding SPECT utility in multiplexed biological investigations [53,54], providing a unique advantage for multiplexed mechanistic studies not readily achievable with PET. Methodological reviews contextualize these advances, underscoring the impact of instrumentation design and reconstruction strategy on achieving robust quantitative SPECT performance [55]. The link between radiopharmaceutical quality control and quantitative imaging output represents a further critical dimension: tracer radiochemistry and QC procedures directly influence the reliability of imaging-derived metrics and must be integrated within quantitative workflows [56]. Cross-calibration between the SPECT system and the dose calibrator, analogous to PET quality control procedures, remains a critical but not yet fully standardized requirement for ensuring absolute quantitative consistency across centers [14,55]. Quality control and harmonization remain the central pillars sustaining quantitative nuclear imaging. Joint guidelines from European Association of Nuclear Medicine (EANM) and European Society for Molecular Imaging (ESMI) formalize structured calibration procedures, rigorous acquisition standards, and transparent analysis workflows for both PET and SPECT, providing the most comprehensive harmonization framework currently available across all preclinical imaging modalities [14]. Ongoing advances in reconstruction algorithms, AI integration, and hybrid imaging architectures continue to enhance both accuracy and interpretability of preclinical nuclear imaging data [1]. Collectively, these developments position quantitative nuclear imaging among the modalities with the most advanced harmonization and calibration infrastructures currently available in preclinical imaging, while PET and SPECT continue to occupy complementary methodological niches characterized by distinct strengths and technical limitations, as discussed in Section 3.3.

### 3.7. Quantitative and Emerging Optical and Photoacoustic Imaging

Optical imaging modalities, including fluorescence, bioluminescence, and photoacoustic imaging, enable quantitative assessment of molecular, cellular, vascular, and functional processes based on photon emission, optical absorption, and acoustic signal detection in biological tissues. Spatial resolution typically ranges from ~1–3 mm in fluorescence-based approaches to ~100–500 μm in photoacoustic imaging, depending on imaging depth and system configuration. Quantitative outputs are strongly influenced by tissue scattering, absorption, light fluence distribution, and reconstruction methodology, requiring model-based calibration and resulting in higher sensitivity to experimental configuration compared with tomographic modalities. Table 6 summarises representative quantitative optical and photoacoustic imaging applications in preclinical research.

The studies included in this section are summarized in Table 6, which provides a structured overview of quantitative optical and photoacoustic imaging applications in preclinical models, detailing the primary quantitative focus, biological model, key quantitative outcome, and level of validated quantitative performance for each included study. Optical imaging modalities offer high molecular sensitivity and compatibility with longitudinal and high-throughput experimental designs, particularly in zebrafish and small-animal models. Their primary metrological challenge, however, is structural: detected photon emissions are heavily influenced by tissue scattering, absorption, and depth-dependent attenuation, rendering quantification intrinsically dependent on explicit photon transport modeling, inverse reconstruction algorithms, and harmonized calibration protocols. Fluorescence signal standardization frameworks address this challenge by defining calibration standards and normalization strategies to reduce system sensitivity biases across instruments [57,58]. Fluorescence Molecular Tomography (FMT) extends planar fluorescence toward volumetric quantification by using diffusion-based models to relate surface measurements to fluorophore distributions, solving an ill-posed inverse problem through regularization and reconstruction constraints. From a theoretical perspective, the quantitative limitation of optical imaging is rooted in the physics of light propagation in highly scattering media. In the diffusion approximation, the mapping between internal fluorophore distributions and boundary measurements can be formulated as a Fredholm integral equation of the first kind, which is inherently ill-posed in the sense of Hadamard [59,60,61]: solutions are non-unique and unstable under measurement noise, requiring explicit regularization for stable inversion. Tikhonov-type and sparsity-promoting approaches are commonly used to stabilise reconstruction, but introduce model-dependent bias linked to assumptions on tissue optical properties. Experimental studies in tissue-mimicking phantoms and small-animal models consistently show that reconstruction accuracy degrades with increasing imaging depth and decreasing optical contrast, leading to depth-dependent localisation errors and systematic biases in fluorophore concentration estimates [59]. Hybrid FMT/Cone Beam CT systems further enhance quantitative accuracy by providing anatomical priors that stabilize inverse solutions [62], while early-photon and compressed sensing approaches improve sensitivity and spatial resolution in preclinical phantom and murine models [63]. Bioluminescence imaging (BLI) provides an alternative quantitative optical approach based on endogenous light emission from enzymatic reactions, with photon emission intensity generally proportional to viable cell density. Automated 3D BLI reconstruction methods mitigate geometric biases by providing volumetric emission estimates [64], while commercial and mobile Bioluminescence Tomography platforms have demonstrated high-precision source localization and robust quantitative performance across murine tumor models [65,66,67]. In addition, in vivo quantitative FRET imaging complements these approaches by enabling dynamic molecular-level readouts, with intensity-based and lifetime-based metrics compared for precision in functional quantification [68]. Standardized injection protocols and consistent imaging conditions are critical prerequisites for maintaining reproducibility across experimental cohorts [69]. Moreover, high-frequency ultrasound (HFUS) represents a complementary quantitative modality, as demonstrated by computer-aided assessment of hepatic steatosis progression in murine models—providing reproducible measurement of liver echogenicity and morphology with potential for non-invasive longitudinal monitoring [70]. Zebrafish imaging exhibits a distinct variance profile within this framework. Technical variance is relatively low in early developmental stages due to optical transparency, but increases with pigmentation and tissue opacity during maturation—so that optical methods, while applicable to larval and early juvenile stages (approximately 0–7 days post-fertilization for maximum transparency, extending in pigmentation-deficient lines such as casper mutants), are not suitable for in vivo imaging of adult fish without fixation and clearing. Biological variance is strongly influenced by rapid developmental dynamics, requiring strict staging and age-matching for quantitative comparability. Computational variance is partially mitigated by the availability of annotated datasets and AI-assisted analysis pipelines, although cross-platform validation remains limited. Overall, zebrafish represents a biologically powerful but metrologically distinct model system that requires stage- and modality-specific validation for quantitative cross-species comparisons. Polarization-sensitive Optical Coherence Tomography (PS-OCT) has been applied in zebrafish tumor xenografts to quantify microstructural birefringence and characterize tumor microenvironmental changes longitudinally [71]. Furthermore, quantitative feature extraction from brightfield and fluorescence imaging supports high-dimensional phenotypic assessment of zebrafish embryos [72], while Mueller matrix OCT combined with deep learning enables structural-functional mapping across developmental stages [73]. High-content automated optical screening platforms further allow multiplexed, volumetric assessment of drug responses and tumor burden at scale [74], and optical microscopy with video-based analysis supports dynamic cardiac functional quantification in zebrafish embryos [75]. In addition, systematic reviews confirm that optical and fluorescence xenograft workflows provide quantitative endpoints suitable for translational tumor research [76], and broader frameworks highlight zebrafish as a versatile model for functional imaging across neurological and developmental applications [77]. On the other hand, photoacoustic imaging bridges the optical and acoustic domains by converting pulsed optical absorption into acoustic signals via thermoelastic expansion, partially mitigating the impact of photon scattering on spatial localization. MSOT frameworks employ spectral unmixing and model-based inversion to estimate physiologically relevant metrics including oxygen saturation (SO_2_) and total haemoglobin concentration (HbT), with depth-dependent fluence correction representing the primary technical challenge [78]. Dual-modality systems combining photoacoustic and fluorescence imaging enable simultaneous structural and molecular quantification in vivo [79,80]. From a cross-modality metrological perspective, optical and photoacoustic imaging occupy a lower level of harmonization maturity compared with nuclear and MRI techniques. Absolute quantification—routinely achievable in PET through calibrated attenuation correction and kinetic modeling, or in CT through the physical traceability of Hounsfield Units—remains fundamentally more challenging in optical imaging due to depth-dependent scattering, tissue heterogeneity, and the ill-posed nature of the optical inverse problem. This limitation is structural rather than purely technical: the forward model linking source distribution to surface measurements is intrinsically underdetermined, and regularization introduces model-dependent bias without direct equivalents in PET or CT. Nevertheless, these modalities exhibit complementary strengths, including superior cross-species applicability (notably in transparent zebrafish models enabling volumetric molecular imaging) and high AI integration readiness driven by the availability of large annotated datasets for deep learning. Photoacoustic imaging partially mitigates these limitations by improving spatial localization through acoustic detection while retaining optical contrast mechanisms. Collectively, optical and photoacoustic modalities occupy a quantitatively distinct domain characterized by high AI integration readiness and cross-species applicability—particularly in zebrafish platforms—but limited harmonization maturity and inherently challenging absolute quantification. Three overarching principles govern quantitative performance in this domain: model fidelity and reconstruction stability, which determine how reliably raw signals map to interpretable biological metrics; bias and variance control, which underpin repeatability and cross-study comparability; and integration with complementary modalities, where optical data anchored by anatomical or functional priors from MRI or nuclear imaging substantially improves quantitative confidence. These principles align directly with the variance control framework outlined in Section 3.3, and their systematic implementation represents the critical pathway toward elevating optical and photoacoustic imaging to the metrological standards achieved by more mature modalities.

## 4. Discussion

Evidence-based conclusions in this Discussion are drawn directly from the reviewed studies and are supported by citations; interpretive statements—including the cross-modality metrological hierarchy, the variance framework synthesis, and the future research roadmap—represent the authors’ analytical synthesis and should be read as structured expert interpretation rather than systematic evidence aggregation.

Quantitative preclinical imaging has evolved from a visualization-driven discipline into a measurement-centered biomarker science in which reproducibility, bias control, and parameter identifiability are as decisive as biological sensitivity. Viewed through the metrological framework established in Section 3.3, differences among imaging modalities reflect not simply contrasts in resolution or molecular sensitivity, but fundamentally distinct balances between model conditioning, acquisition stability, and harmonization maturity. This interpretive lens reveals a clear hierarchy of quantitative readiness across modalities—one with direct practical implications for study design, biomarker selection, and translational applicability [24,28].

A key contribution of this review is the three-dimensional analytical framework developed in Section 3.3—articulating technical, biological, and computational variance as analytically separable dimensions—which provides the interpretive lens through which the modality-specific findings discussed below should be read. Importantly, these three dimensions are not independent but form a coupled system in which improvements in one domain directly influence the others. For instance, harmonized acquisition and reconstruction protocols reduce technical variance, thereby enhancing the reliability of biological validation, while robust validation frameworks can in turn reveal latent sources of bias in the imaging pipeline.

Nuclear imaging, particularly PET, provides inherently quantitative measurements with well-established calibration frameworks and a relatively mature harmonization infrastructure, supporting improved reproducibility and cross-platform comparability. Multicenter standardization initiatives within the EANM framework have demonstrated that controlled reconstruction settings and cross-system calibration significantly reduce SUV variability and improve inter-platform concordance [1,16,81]. Nevertheless, static SUV remains a simplified surrogate of tracer kinetics, and dynamic PET—while enabling compartmental modeling and estimation of physiologically meaningful rate constants—amplifies sensitivity to arterial input function errors, statistical noise, and parameter identifiability limitations [17,19]. Independent radiopharmaceutical assays remain necessary to validate that quantitative imaging metrics accurately reflect underlying biological processes [82]. SPECT has similarly progressed through advances in attenuation and scatter correction and multi-pinhole collimation [83], yet remains comparatively constrained by count statistics and calibration sensitivity relative to PET. The application of PET beyond murine systems—illustrated by its implementation in adult zebrafish [3]—further demonstrates both scalability and methodological fragility: metrological validation must be explicitly re-established whenever biological scale or acquisition geometry changes, a principle that applies equally across all modalities.

Shifting from nuclear to structural imaging, CT and micro-CT provide attenuation-based quantification traceable to physical units, with reproducible structural and densitometric metrics validated across skeletal, pulmonary, and vascular applications when workflows are harmonized [8,30,31]. Cross-platform accreditation frameworks remain less developed than those for PET, and longitudinal applications require dose optimization to avoid biological confounding [9].

Where CT provides structural specificity, MRI offers unmatched multiparametric depth without ionizing radiation, but its harmonization infrastructure—while advancing through ISMRM consensus recommendations [6,26,84]—remains constrained by vendor heterogeneity and the absence of universally adopted phantom standards, representing its primary metrological challenge relative to PET.

Beyond the electromagnetic spectrum, Optical and photoacoustic imaging offer high molecular sensitivity and throughput, particularly in zebrafish platforms, but face structural metrological limitations from ill-posed inverse problems and underdeveloped harmonization infrastructure [85]. Photoacoustic imaging partially mitigates quantification challenges through acoustic detection, yet consensus calibration protocols remain emerging rather than established [78,86]. Compared with PET and MRI, standardized cross-platform calibration and accreditation infrastructures for photoacoustic systems are still limited, and consensus protocols for quantitative validation are emerging rather than established [27].

Taken together, these modality-specific considerations converge on three structural challenges that cut across all quantitative preclinical imaging frameworks and remain incompletely resolved. The first is the absence of shared biological reference standards for cross-modality validation. While technical harmonization has advanced substantially—particularly in nuclear imaging—biological validation frameworks that anchor imaging-derived parameters to independent molecular or histological endpoints remain inconsistently implemented across modalities and laboratories. This gap is particularly consequential for translational research, where the biological interpretability of imaging biomarkers is as important as their technical reproducibility [24,28]. The second challenge is cross-species parameter transferability. Quantitative strategies validated in murine models cannot be assumed to retain metrological performance in zebrafish, rabbit, or larger animal systems without explicit re-validation—a requirement that is frequently overlooked in translational study designs [3,5]. The third challenge is the validation of AI-assisted components within quantitative pipelines. AI-driven approaches are increasingly integrated into preclinical imaging workflows, enabling automated segmentation, feature extraction, and parameter estimation across modalities, with the potential to reduce operator-dependent variability and enhance sensitivity in complex datasets [83,87]. However, the incorporation of artificial intelligence introduces an additional layer of methodological complexity. Quantitative outputs become dependent not only on acquisition parameters but also on model architecture, training strategies, and preprocessing pipelines, resulting in an inherent algorithmic dependency that must be explicitly addressed. A critical limitation lies in the sensitivity of AI models to domain shift, whereby performance may degrade when applied to data acquired under different experimental conditions, imaging systems, or biological models. This issue is closely linked to training bias, as model performance is strongly influenced by the representativeness of the training dataset. Consequently, the generalizability of AI-derived quantitative metrics across laboratories and study designs remains uncertain.

A particularly consequential challenge for AI-assisted quantitative pipelines is model generalization under dataset shift. Models trained on data originating from a single center, acquisition protocol, scanner generation, or animal handling workflow may fail to preserve quantitative accuracy when deployed across heterogeneous experimental environments. In preclinical imaging, this limitation is amplified by inter-laboratory variability in anesthesia protocols, physiological monitoring, acquisition settings, and annotation practices. Dataset bias arising from unbalanced training distributions or inconsistent reference annotations may therefore introduce systematic errors into derived quantitative parameters that remain difficult to detect without dedicated out-of-distribution validation. Accordingly, validation frameworks for AI-assisted quantitative imaging should prospectively incorporate multi-site, multi-protocol, and temporally independent testing, together with explicit reporting of performance degradation under distributional shift.

Beyond generalization and dataset shift considerations already discussed, deep learning introduces additional metrological constraints related to explainability, robustness, and translational readiness. Explainability remains limited, as deep neural networks operate as high-capacity function approximators whose internal representations are not directly interpretable in physical or biological terms, restricting traceability of quantitative outputs. Robustness is affected by performance degradation under distributional shifts arising from differences in acquisition protocols, scanner hardware, reconstruction pipelines, and biological variability. Finally, regulatory and translational deployment of AI-assisted quantitative pipelines requires prospective validation of accuracy, precision, and stability under representative conditions, which is not yet systematically established in preclinical imaging. Collectively, these factors highlight that metrological credibility of AI-based imaging depends on interpretability, robustness, and validation frameworks aligned with translational requirements.

A structural limitation affecting reproducibility in preclinical quantitative imaging is the scarcity of shared reference datasets and community benchmarking frameworks. Unlike clinical imaging, where initiatives such as The Cancer Imaging Archive (TCIA) support standardized algorithm evaluation and cross-study comparability, preclinical imaging currently lacks equivalent large-scale infrastructure. Within the reviewed literature, publicly available benchmark datasets remain limited; a notable example is an annotated whole-body micro-CT dataset of tumor-bearing mice with multi-annotator segmentation, which provides a partial reference standard for CT-based applications. For MRI, PET, and emerging hybrid modalities, the absence of shared phantom datasets and harmonized ground-truth references limits cross-platform calibration and independent validation of quantitative pipelines.

This lack of external validation constrains the assessment of robustness and may lead to overestimation of model performance. As a result, while AI-based methods show considerable promise for improving quantitative imaging pipelines, their metrological credibility depends on rigorous validation, transparency in model development, and systematic evaluation across heterogeneous experimental conditions.

The limitations of this comparative synthesis—including heterogeneity in quantitative definitions across disciplines, incomplete validation against independent reference standards in a proportion of included studies, and the methodological constraints inherent to a structured narrative review design—are discussed in detail in the Limitations of This Review section.

**Figure 4:**
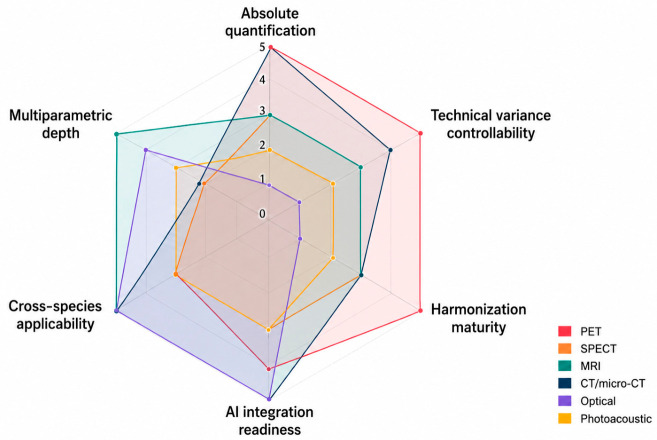
Quantitative performance profiles of preclinical imaging modalities across six metrological dimensions. Each modality is scored on a qualitative scale from 1 (absent or very limited) to 5 (consolidated and internationally validated), based on systematic evaluation of the reviewed evidence according to predefined scoring criteria (see Supplementary Table S1). The radar plot is intended as an integrated visual summary of the comparative analysis developed in Section 3.3, Section 3.4, Section 3.5, Section 3.6 and Section 3.7 and should be read in conjunction with Table 2.

Addressing these challenges collectively requires moving beyond modality-specific optimization toward a convergence of complementary strengths within shared metrological standards and multimodal validation architectures. The quantitative performance profile of each modality—as illustrated in Figure 4—makes this complementarity explicit.

PET’s strength in absolute quantification and harmonization maturity, MRI’s depth in multiparametric tissue characterization, and optical imaging’s scalability and molecular sensitivity are not competing attributes but potentially synergistic ones, if integration is anchored in biological co-validation rather than mere technical co-registration. Concrete evidence of quantitative improvement through multimodal integration is available within the reviewed literature. First, the combination of FMT with cone-beam CT priors (Section 3.7) demonstrates that anatomical constraints reduce inverse problem instability in optical reconstruction, improving fluorophore localization accuracy and reducing depth-dependent bias that cannot be mitigated within an optical-only framework [60,62]. Second, 3D MR Fingerprinting illustrates how multiparametric integration within a single modality can reduce acquisition time while preserving quantitative fidelity—a principle analogous to, though distinct from, true cross-modality integration, and directly relevant to longitudinal preclinical studies requiring repeated measurements under anesthesia constraints [20]. A further example of genuine cross-modality quantitative improvement is provided by dual-modality photoacoustic and fluorescence systems (Section 3.7), where simultaneous acquisition of SO_2_ and molecular fluorescence readouts provides complementary biological constraints that reduce ambiguity in the interpretation of either signal alone [79,80]. These examples illustrate that multimodal integration improves quantitative performance by introducing complementary constraints that reduce underdetermination and variance in the primary measurement pipeline. Hybrid platforms such as PET/MRI already demonstrate this principle in practice, enabling simultaneous acquisition of complementary quantitative readouts while introducing additional layers of technical complexity that themselves require structured quality assurance [4,5]; standardized computational pipelines for automated feature extraction and image quantification have been proposed to improve reproducibility and reduce operator-dependent variability [88,89]. Conversely, readers specifically interested in radiomics-based quantification approaches in preclinical imaging may refer to [90] for a dedicated and in-depth treatment of methodological advances and emerging applications in that domain.

On balance, the future of preclinical imaging lies in multimodal integration anchored in shared metrological standards, AI frameworks constrained by interpretability, robustness, and validation safeguards, and cross-scale validation strategies aligning molecular, microstructural, and organism-level readouts within a coherent quantitative architecture. Translational impact will depend less on incremental gains in resolution and more on the stability, transparency, and biological validity of quantitative pipelines maintained across platforms, laboratories, and experimental models.

Based on the analytical synthesis presented in this review, we identify five priority research directions for quantitative preclinical imaging: (1) extension of harmonization infrastructure beyond PET/SPECT to MRI, CT, and optical modalities through coordinated phantom standards and multicenter calibration studies; (2) establishment of AI-assisted quantitative imaging as a metrologically validated pipeline component, including explainability and robustness assessment; (3) development of shared datasets and benchmarking frameworks for reproducible evaluation; (4) systematic cross-species re-validation of quantitative strategies; and (5) development of multimodal co-validation frameworks anchored in shared biological reference standards. Progress in these areas will require coordinated advances across imaging physics, computational methodology, and biological validation.

### Limitations of This Review

Despite the structured approach adopted, several limitations should be acknowledged. This work follows a structured narrative review design rather than a full systematic review with formal risk-of-bias assessment, reflecting the heterogeneity and metrological focus of the included literature. Consequently, no study-level quality scoring was performed. In addition, the literature search was restricted to major scientific databases, which may have introduced selection bias and limited coverage of grey literature. The distribution of studies across imaging modalities was uneven, with a relative predominance of nuclear and MRI-based approaches compared with optical and emerging hybrid techniques, potentially influencing comparative interpretations. Furthermore, the definition of quantitative imaging is inherently heterogeneous across disciplines, encompassing physically calibrated measurements, model-based parameters, and biologically validated biomarkers; variability in methodological implementations and validation strategies across studies therefore limits direct comparability of results. A proportion of the included studies additionally lacked comprehensive validation against independent reference standards, reflecting a broader limitation in the field related to the availability of robust ground truth data in preclinical settings. Finally, increasing integration of AI-based methodologies introduces additional variability linked to training data composition and generalisation performance across experimental conditions. These limitations should be considered when interpreting the comparative metrological framework and its translational implications.

## 5. Conclusions

Quantitative preclinical imaging is no longer defined solely by technological innovation but by its capacity to generate reproducible, bias-controlled, and biologically validated metrics across experimental systems and biological models. The framework developed in this review—distinguishing technical, biological, and computational sources of variance as analytically separable dimensions of quantitative uncertainty—reveals that the modalities considered here do not occupy a uniform level of metrological readiness. Nuclear imaging, particularly PET, currently offers the most mature harmonization infrastructure and the most traceable absolute quantification capability; MRI provides unparalleled multiparametric depth and cross-species flexibility; CT and micro-CT deliver physically traceable structural metrics with increasingly mature AI-assisted pipelines; optical and photoacoustic modalities afford high-throughput molecular sensitivity and scalability, at the cost of inherently ill-posed quantification problems and underdeveloped harmonization frameworks. These distinct profiles carry direct implications for study design and translational strategy. Selecting a quantitative imaging modality should be guided not only by biological sensitivity but by the metrological maturity of the available validation infrastructure, the controllability of variance sources within the experimental context, and the availability of harmonized protocols that permit cross-platform and cross-species comparability. Where single modalities are insufficient, multimodal integration anchored in biological co-validation—rather than mere technical co-registration—offers the most promising pathway toward robust and translationally relevant biomarker generation. The next phase of progress will depend on three converging developments: the extension of harmonization infrastructure to MRI, CT, and optical modalities through internationally adopted standards; the prospective validation of AI-assisted components as metrologically credible elements of quantitative pipelines; and the systematic implementation of cross-species re-validation strategies whenever biological scale or acquisition geometry changes. Ultimately, the translational value of preclinical imaging will be determined not by signal intensity or spatial resolution alone, but by the robustness, comparability, and physiological interpretability of the quantitative biomarkers it produces—and by the transparency of the metrological frameworks that underpin them.

## Figures and Tables

**Table 1:** Classification of studies.

Database	PubMed	Scopus	Web of Science		
**Period**	2015–2026				
**Model**	Murine	Rabbit	Zebrafish	Small Animals	
**Modality**	CT	MRI	PET/SPECT	Optical	Photoacoustic
**Purpose**	Prediction	Bio- correlation	Toxicology		
**Study Type**	Original	Reviews			

**Table 2:** Comparative quantitative performance of preclinical imaging modalities across key metrological dimensions. Scores reflect a qualitative assessment based on systematic evaluation of the reviewed literature (see Supplementary Table S1). High: well-established performance; Medium: partially established; Low: limited or technically challenging.

Modality	Absolute Quantification	Technical Variance Controllability	Harmonization Maturity	AI Integration Readiness	Cross-Species Applicability	Multiparametric Depth
**PET**	High	High	High	High	Medium	Low
**SPECT**	Medium	Medium	Medium	Medium	Medium	Low
**MRI**	Medium	Medium	Medium	High	High	High
**CT/micro-CT**	High	High	Medium	High	High	Low
**Optical**	Low	Low	Low	High	High	High
**Photoacoustic**	Low	Low	Low	Medium	Medium	Medium

**Table 3:** Overview of quantitative CT studies.

Study	Year	Focus	Model	Key Quantitative Outcome	Quantitative Performance
Clark et al. [8]	2021	Advanced micro-CT technologies: attenuation calibration, material decomposition, and automated segmentation	-Review -Small Animal	Multi-parameter structural metrics—Hounsfield Unit-based quantification, material-specific attenuation coefficients	High—comprehensive methodological framework; calibration and reconstruction standards reviewed
Christiansen et al. [29]	2016	Effect of voxel size and segmentation method on trabecular bone microstructure metrics	Murine	Bone Volume to Total Volume; (BV/TV), Trabecular Thickness (Tb.Th), Trabecular Separation; (Tb.Sp); trabecular architecture descriptors sensitive to acquisition and segmentation parameters	Medium—systematic parameter sensitivity analysis; reproducibility dependent on protocol consistency
Oliviero et al. [30]	2022	Reproducibility of densitometric and biomechanical metrics from in vivo micro-CT tibia images	Murine	Bone Mineral Density (BMD), cortical thickness, stiffness estimates—longitudinal skeletal biomarkers	High—repeatability and reproducibility coefficients formally reported; in vivo validation
Ferrini et al. [31]	2025	Longitudinal micro-CT for quantitative assessment of pulmonary disease in small animals	Small Animal	Total lung volume, aerated lung fraction, mean lung density—structural pulmonary biomarkers	High—standardized longitudinal workflow validated; quantitative descriptors reproducible across timepoints
Vincenzi et al. [32]	2022	Fully automated deep learning pipeline for micro-CT densitometry in pulmonary fibrosis models	Murine	Lung density distribution—AI-derived densitometric descriptors with histopathological correlation	High—automated pipeline validated against manual annotations; deep learning segmentation accuracy reported
Buccardi et al. [33]	2023	Fully automated micro-CT deep learning approach for lung fibrosis progression and therapy response	Murine	Lung fibrosis extent, density metrics—longitudinal therapy response indicators	High—prospective validation; automated quantification compared to expert assessment
Cheng et al. [34]	2025	AI-assisted semiquantitative measurement of bleomycin-induced lung fibrosis using in vivo micro-CT	Murine	Fibrosis score, lung density—end-to-end AI-assisted quantification pipeline	Medium—AI-assisted approach validated; semiquantitative rather than fully quantitative output
Jensen et al. [35]	2024	Annotated whole-body micro-CT database of subcutaneous tumors with multi-annotator segmentation	Murine	Tumor volume, segmentation consistency metrics—inter-observer reproducibility benchmarks	High—multi-annotator dataset enables reproducibility benchmarking; publicly available reference standard
Brown et al. [9]	2024	Comparative analysis of preclinical CT radiomics using cone-beam and micro-CT scanners	Murine	Radiomic features, reproducibility metrics—cross-platform feature stability assessment	Medium—cross-platform reproducibility demonstrated; feature stability highly dependent on acquisition and reconstruction harmonization
Pereira-Rosa et al. [36]	2024	Non-invasive skeletal muscle quantification in small animals using micro-CT	Protocol (JoVE) -Small Animals	Muscle volume, cross-sectional area—morphometric descriptors for musculoskeletal assessment	Medium—methodology demonstrated; formal reproducibility metrics not fully reported
Ashton et al. [37]	2015	In vivo micro-CT with nanoparticle contrast agents for vascular and perfusion imaging	-Review -Small Animals	Vascular volume fraction, contrast distribution—semi-quantitative vascular architecture metrics	Medium—contrast-enhanced vascular quantification demonstrated; absolute quantification limited by agent pharmacokinetics

**Table 4:** Overview of quantitative MRI studies.

Study	Year	Focus	Model	Key Quantitative Outcome	Quantitative Performance
Jelescu et al. [6]	2025	Diffusion Weighted Imaging (DWI)—Apparent Diffusion Coefficient (ADC) quantification; ISMRM acquisition and modeling guidelines for quantitative robustness	Guidelines Small Animals	ADC—structured recommendations for gradient calibration, motion management, and b-value selection	High—consensus-based framework; reproducibility benchmarks defined
Albrecht et al. [38]	2019	DWI—ADC quantification in healthy and tumor tissues on preclinical PET/MRI platform	Rats	ADC—compatibility with hybrid PET/MRI acquisition confirmed	Medium—single-center validation; cross-platform reproducibility not assessed
Duan et al. [39]	2024	Intravoxel Incoherent Motion;/Diffusion Kurtosis Imaging (IVIM/DKI)—separation of molecular diffusion and microvascular perfusion components	Murine	Slow Diffusion Coefficient (D), Fast Diffusion Coefficient (D*), Perfusion Fraction (f)—(IVIM); Diffusion Kurtosis (K), D (DKI)—hypoxia biomarker correlation with histological endpoints	Medium—biological validation available; inter-session reproducibility not reported
Guo et al. [40]	2022	IVIM/DKI—monitoring radiotherapy response through perfusion-sensitive and microstructural parameters	Rabbit	D, D*, f, K—therapy response indicators with histopathological correlation	Medium—longitudinal design; single-center; limited reproducibility metrics
Pickup et al. [41]	2022	Dynamic Contrast-Enhanced MRI; (DCE-MRI)—optimized quantitative protocol for preclinical cancer models; arterial input function (AIF) estimation	Murine	Ktrans, ve—vascular permeability and extravascular volume fraction	High—optimised AIF strategy; protocol reproducibility validated in vivo
Zhu et al. [20]	2025	MRI Fingerprinting—simultaneous T1/T2 mapping with dynamic contrast acquisition in single scan	Murine	T1, T2, DCE parameters—simultaneous multiparametric estimation	High—dictionary-based validation; simultaneous parameter estimation confirmed in vivo
Roudi et al. [42]	2024	Relaxometry—T2/T2* mapping; edema, fibrosis, and tumor microenvironment assessment	Murine	T2, T2*—between-session variability quantified under controlled conditions	Medium—repeatability coefficients reported; biological and technical variance partially separated
Wei et al. [43]	2016	Quantitative Susceptibility Mapping (QSM)—tissue magnetic susceptibility for iron deposition, hemorrhage, and calcification	Murine	chi (susceptibility)—iron deposition and hemorrhage quantification	Medium—methodology validated; preclinical-specific reproducibility data limited
Herrman et al. [44]	2016	^1^H-MRSwith T1 mapping—metabolic quantification combined with longitudinal relaxation assessment	Murine	Metabolite concentrations, T1—combined metabolic and structural characterization	Medium—multiparametric combination demonstrated; reproducibility metrics not fully reported

**Table 5:** Overview of quantitative nuclear medicine studies.

Study	Year	Focus	Model	Key Quantitative Outcome	Quantitative Performance
Kuntner et al. [17]	2024	Harmonization of Standardized Uptake Value (SUV) acquisition and analysis to reduce variability and improve reproducibility	Small Animals	SUV—multicentric variability reduction through standardized acquisition and analysis	High—multicentric study; SUV variability formally quantified and reduced through harmonized protocols
Knyzeliene et al. [45]	2024	Dynamic PET quantification using compartmental and graphical kinetic modeling	Small Animals	Ki, k1-k4, Distribution Volume Ratio (DVR)—kinetic rate constants from compartmental and graphical modeling	Medium—kinetic modeling framework validated; AIF sensitivity acknowledged
Kuttner et al. [46]	2024	AI-assisted arterial input function estimation to improve kinetic parameter accuracy	Murine	AIF-derived kinetic parameters—AI-assisted estimation reduces operator dependency	Medium—AI-AIF approach validated in vivo; generalizability across tracers not fully established
Pavone et al. [47]	2024	PET as a tool to identify quantitative biomarkers in preclinical imaging	Murine	SUV, metabolic rate—imaging biomarker identification and biological validation	Medium—biomarker framework proposed; multicentric validation not reported
Mannheim et al. [3]	2025	Cross-platform quantitative comparison between preclinical and clinical total-body PET/CT	Small Animals	SUV, partial volume effects—cross-platform and cross-scale quantitative concordance	Medium—translational concordance demonstrated; scale-dependent challenges explicitly reported
Raccagni et al. [48]	2018	Evaluation of response to neo-adjuvant chemotherapy in a triple-negative breast cancer (TNBC) murine model	Murine	PET—SUV—tumor metabolic activity quantification	Medium—biological validation with histological endpoints; single-center study
Gargiulo et al. [49]	2017	PET molecular imaging of neuroinflammation; quantitative microglial activation assessment	-Review -Rodents	Binding Potential, SUV—neuroinflammatory biomarker quantification	Medium—biological validation with histological correlation; single-center
Benfante et al. [50]	2022	PET biodistribution analysis of 64Cu-chelator radiotracer in murine models	Murine	SUV, organ-specific uptake—radiotracer biodistribution and biological validation	Medium—biological validation available; quantitative reproducibility not formally reported
Tucker et al. [51]	2021	Radiotracer uptake quantification and longitudinal biodistribution in zebrafish	Zebrafish	SUV, whole-body biodistribution—cross-species feasibility of quantitative nuclear imaging	Limited—proof-of-concept; pronounced partial volume effects; metrological re-validation required
Gerdekoohi et al. [52]	2017	Absolute activity quantification with attenuation correction and iterative reconstruction in SPECT	Small Animals	Absolute activity concentration—attenuation-corrected quantification with calibration factors	Medium—absolute quantification demonstrated; calibration stability not longitudinally assessed
Lukas et al. [53]	2020	Quantitative validation of simultaneous multi-radionuclide small-animal SPECT imaging	Small Animals	Activity concentration per radionuclide—simultaneous multi-isotope quantitative validation	High—simultaneous multi-radionuclide validation; quantitative integrity formally assessed
Prieto et al. [54]	2022	Multi-isotope quantitative validation in preclinical SPECT/CT	Small Animals	Activity concentration, recovery coefficients—multi-isotope SPECT/CT quantitative accuracy	High—multi-isotope validation with recovery coefficient analysis; cross-calibration reported
Enninful et al. [55]	2026	Review of SPECT instrumentation and reconstruction strategies impacting quantitation	Review	Quantitative accuracy metrics—instrumentation and reconstruction impact on SPECT quantification	High—comprehensive methodological review; reconstruction and collimator impact systematically analysed
Altunay et al. [56]	2024	PET tracer radiochemistry and Quality Control (QC) linked to quantitative imaging workflows	-Protocol (JoVE) -Small Animals	Radiochemical purity, specific activity—radiopharmaceutical QC impact on quantitative PET	Medium—radiochemical QC framework described; direct link to imaging reproducibility partially established
Vanhove et al. [14]	2024	Quality control and harmonization standards for quantitative PET and SPECT	-Guidelines -Small Animals	QA framework—structured calibration, acquisition standards, and analysis workflows for PET/SPECT	High—international guideline; EANM/ESMI endorsed; formally adopted quality assurance framework
Bruzgo-Grzybko et al. [1]	2025	Comprehensive review on quantitative performance and translational relevance of PET/SPECT	-Review -Small Animals	Translational performance metrics—synthesis of quantitative PET/SPECT capabilities and limitations	High—comprehensive evidence synthesis; multicentric and translational perspective

**Table 6:** Overview of Preclinical Quantitative and Emerging Optical and Photoacoustic Imaging.

Study	Year	Focus	Model	Key Quantitative Outcome	Quantitative Performance
Refaat et al. [57]	2022	Fluorescence imaging standardization, calibration, and reproducibility frameworks	Review	Signal calibration standards—reproducibility requirements for quantitative fluorescence imaging	Medium—standardization framework proposed; formal validation across platforms limited
Chu et al. [58]	2025	Optical imaging harmonization, workflow standardization, cross-lab reproducibility	Review	Cross-lab reproducibility metrics—harmonization strategies for quantitative optical imaging	Medium—harmonization framework outlined; multicentric adoption not yet established
Lo et al. [59]	2020	Fluorescence Diffuse Optical Tomography with ultrasound priors; reconstruction accuracy	Small Animals	Fluorophore concentration maps—depth-corrected volumetric fluorescence quantification	Medium—reconstruction accuracy improved with anatomical priors; absolute quantification partially validated
An et al. [60]	2018	Fluorescence Molecular Tomography (FMT); inverse problem regularization	Review	Volumetric fluorophore distribution—regularized inverse solution for FMT quantification	Medium—reconstruction methodology validated in phantoms; in vivo reproducibility limited
Zhang et al. [61]	2022	FMT reconstruction; diffusion-based modeling, volumetric quantification	Review	Volumetric fluorophore concentration—diffusion model-based FMT reconstruction	Medium—modeling framework validated; sensitivity to optical property assumptions acknowledged
Nouizi et al. [62]	2022	Hybrid FMT/Cone Beam Computed Tomography (CBCT); anatomical priors to improve inverse solution stability	Small Animals	Fluorophore concentration—anatomically constrained FMT reconstruction with improved stability	Medium—hybrid approach improves quantitative stability; cross-platform validation not reported
Konovalov et al. [63]	2021	Early-photon and compressed sensing; sensitivity and spatial resolution improvement	Small Animals	Spatial resolution, sensitivity metrics—early-photon approach for improved optical quantification	Limited—proof-of-concept in phantoms; in vivo quantitative validation limited
Klose et al. [64]	2018	3D Bioluminescence imaging (BLI); volumetric reconstruction, geometric bias correction	Small Animals	Volumetric bioluminescence emission—3D BLI reconstruction with geometric bias reduction	Medium—volumetric reconstruction validated; substrate delivery variability acknowledged
Thompson et al. [65]	2023	Dual-modality photoacoustic and fluorescence imaging; dynamic perfusion quantification	Murine	Oxygen Saturation (SO_2_), Total Hemoglobin (HbT), fluorescence signal—simultaneous structural and molecular dynamic quantification	Medium—dual-modality quantification demonstrated; reconstruction stability partially validated
Deng et al. [66]	2022	Mobile Bioluminescence Tomography (BLT); source localization and reconstruction accuracy	Small Animals	Source position, bioluminescence intensity—volumetric source localization accuracy	Medium—high-precision source localization demonstrated; cross-system reproducibility not reported
Xu et al. [67]	2023	Commercial BLT platform; quantitative accuracy and performance assessment	Small Animals	Bioluminescence flux, source reconstruction accuracy—commercial BLT quantitative performance	Medium—commercial platform performance assessed; biological variability sources acknowledged
Smith et al. [68]	2023	In vivo quantitative Förster Resonance Energy Transfer (FRET); intensity vs. lifetime analysis for molecular interactions	Small Animals	FRET efficiency, donor lifetime—dynamic molecular interaction quantification	Medium—intensity vs. lifetime comparison validated; depth-dependent limitations acknowledged
Kim et al. [69]	2022	Standardized injection protocols; reproducibility in optical imaging experiments	Review	Signal reproducibility metrics—injection protocol standardization impact on optical quantification	Medium—standardization recommendations provided; formal multicentric validation absent
Gargiulo et al. [70]	2025	Quantitative High-Frequency Ultrasound (HFUS) assessment of hepatic steatosis in mice using computer-aided analysis	Murine	Liver echogenicity, morphology metrics—reproducible longitudinal HFUS quantification	Medium—computer-aided analysis validated; cross-scanner reproducibility not assessed
Lichtenegger et al. [71]	2022	Polarization-Sensitive Optical Coherence Tomography (PS-OCT) in zebrafish tumor xenografts; microstructural birefringence quantification	Zebrafish	Birefringence, retardation maps—polarimetric microstructural metrics for tumor characterization	Medium—quantitative polarimetric metrics validated in zebrafish; longitudinal reproducibility limited
Bini et al. [72]	2024	Brightfield imaging; high-dimensional feature extraction and texture quantification in zebrafish	Zebrafish	Morphometric and texture features—radiomics-based quantitative phenotyping	Medium—feature extraction pipeline validated; test–retest reproducibility not formally reported
Li et al. [73]	2023	Mueller matrix Optical Coherence Tomography (OCT) with deep learning; structural-functional mapping in zebrafish	Zebrafish	Polarimetric tissue properties—deep learning-assisted structural-functional quantification	Medium—deep learning integration demonstrated; generalizability across developmental stages limited
Sturtzel et al. [74]	2025	High-content automated optical microscopy; multiplexed phenotypic quantification	Zebrafish	Volumetric tumor burden, phenotypic metrics—automated multiplexed quantification	High—high-content automated platform; multiplexed quantification validated across cohorts
Mitovic et al. [75]	2025	Optical microscopy for dynamic cardiac functional quantification in zebrafish	Zebrafish	Heart rate, contractility metrics—motion-tracking-based cardiac functional quantification	Medium—functional quantification validated; inter-session reproducibility not reported
Cani et al. [76]	2026	Optical/fluorescence xenograft workflows; tumor burden quantification in zebrafish	Zebrafish	Tumor burden, fluorescence intensity—reproducible endpoint harmonization in xenograft models	Medium—systematic review; endpoint harmonization strategies outlined; reproducibility data variable across included studies
Turrini et al. [77]	2023	Functional fluorescence imaging; dynamic neural activity quantification in zebrafish	Zebrafish	Neural activity maps, spatiotemporal signal metrics—functional fluorescence quantification	Medium—functional imaging framework validated; quantitative reproducibility across preparations limited
Upputuri et al. [78]	2016	Multispectral photoacoustic imaging (MSOT); spectral unmixing and fluence correction	Review	SO_2_, HbT—oxygen saturation and total haemoglobin quantification	Limited—methodology reviewed; depth-dependent fluence correction remains a key unresolved limitation
Humbert et al. [79]	2020	Photoacoustic vs. fluorescence tomography; physiologically relevant metric comparison	Murine	Fluorophore concentration, photoacoustic signal—cross-modality quantitative comparison	Medium—comparative validation available; absolute quantification limited by fluence heterogeneity
Sun et al. [80]	2024	Dual-modality photoacoustic and fluorescence imaging; structural and molecular quantification	Small Animals	SO_2_, HbT, fluorescence signal—simultaneous dual-modality dynamic quantification	Medium—dual-modality approach validated; cross-session reproducibility not formally assessed

## Data Availability

The original contributions presented in this study are included in the article. Further inquiries can be directed to the corresponding author.

## Supplementary Materials

The following supporting information can be downloaded at: https://www.mdpi.com/article/10.3390/jimaging12060242/s1, Table S1: Scoring rubric and justification for the assessment of quantitative performance across preclinical imaging modalities.; Table S2: Quantitative performance ranges for preclinical imaging modalities, Table S3: Modality scores with justification and key references.
