# Supplementary material for: Quantitative Preclinical Imaging as a Metrological Framework: Reproducibility, Validation, and Translational Maturity
Source: J Imaging. 2026 May 29;12(6):242. doi: 10.3390/jimaging12060242 (PMC13301819; doi:10.3390/jimaging12060242)
Supplement: Supplementary file 1 [file jimaging-12-00242-s001.zip › jimaging-4227486-supplementary.pdf]

Supplementary Materials

A Comprehensive Review of Quantitative Preclinical Imaging: Methods, Validation, and Translational Integration

Nicolò Lauciello, Giorgio Russo, Alessandro Stefano

Supplementary Table S1. Scoring rubric and justification for the assessment of quantitative performance across preclinical imaging modalities.

Part A. Predefined scoring rubric

The following rubric defines the scoring criteria applied to each modality across six metrological dimensions. Scores range from 1 (absent or very limited) to 5 (consolidated and internationally validated). Scores reflect the current state of evidence in the reviewed literature rather than intrinsic physical limitations of each modality and may evolve as harmonization infrastructure and validation frameworks develop.

Dimension	Score	Criterion
**Absolute quantification capability**	**1**	Qualitative or semi-quantitative measures only; no traceability to reference units
	**2**	Relative quantification possible but dependent on strong modelling assumptions
	**3**	Absolute quantification achievable under controlled conditions but with relevant systematic uncertainty
	**4**	Absolute quantification consolidated for specific applications with available biological validation
	**5**	Absolute quantification traceable to physical or biological units; validated in multicentre studies
**Technical variance controllability**	**1**	Technical variance sources poorly characterized; no standardized control procedures available
	**2**	Some variance sources identified but control strategies limited or unvalidated
	**3**	Control procedures available for main variance sources but not universally adopted
	**4**	Validated control strategies available for most variance sources
	**5**	Complete QA framework with standardized phantoms and multicentre-validated procedures
**Harmonization maturity**	**1**	No international guidelines; no accreditation framework available
	**2**	Emerging guidelines proposed by single groups; not internationally adopted
	**3**	Consensus recommendations available for some applications; partial adoption
	**4**	International guidelines consolidated for most applications; accreditation framework in development
	**5**	Complete international framework with formal accreditation; adopted by major scientific societies

Dimension	Score	Criterion
**AI integration readiness**	**1**	AI applications absent or in purely exploratory phase
	**2**	AI approaches proposed but with limited validation on preclinical data
	**3**	AI approaches validated for specific applications; not generalizable cross-platform
	**4**	AI approaches validated for multiple applications; pipeline integration ongoing
	**5**	AI fully integrated in quantitative pipelines with prospective and cross-platform validation
**Cross-species applicability**	**1**	Applicable to a single species; transferability not demonstrated
	**2**	Applied to multiple species without formal metrological re-validation
	**3**	Applied to multiple species with partial validation; scale limitations known but unresolved
	**4**	Applied to multiple species with metrological validation available for main combinations
	**5**	Consolidated cross-species validation framework; scalability demonstrated with formal re-validation
**Multiparametric depth**	**1**	Only one primary quantitative parameter extractable
	**2**	Two parameters extractable but with mutual modelling dependency
	**3**	Multiple parameters extractable with distinct protocols; limited integration
	**4**	Multiple parameters extractable in combined protocols with partial validation
	**5**	Consolidated multiparametric framework with simultaneous validation of biologically independent parameters

Supplementary Table S2. Quantitative performance ranges for preclinical imaging modalities.

Values represent typical ranges reported in the reviewed literature for dedicated small-animal imaging systems under optimized acquisition conditions. Actual performance is system-, protocol-, and application-dependent. Spatial resolution values refer to in vivo imaging unless otherwise indicated. Sensitivity values for nuclear modalities refer to detection limits in solution; MRI sensitivity is protocol-dependent and not directly comparable. Values should be interpreted as indicative benchmarks rather than absolute specifications.

Modality	Spatial Resolution	Sensitivity / Detection Limit	Main Quantitative Metrics
**PET**	~1–2 mm FWHM (dedicated small-animal systems)	~10^{-11}–10^{-12} mol/L	SUV, kinetic parameters (Ki, k1–k4, DVR, BPND)
**SPECT**	~0.5–1.5 mm (multi-pinhole collimation)	~10^{-10}–10^{-11} mol/L	Absolute activity concentration, multi-radionuclide uptake
**MRI**	~50–200 µm in vivo (field-strength and coil dependent)	Protocol-dependent; mM range for spectroscopic detection	ADC, Ktrans, ve, T_1, T_2, T_2^*, χ (susceptibility), metabolite concentrations
**CT / micro-CT**	~5–50 µm (ex vivo micro-CT); ~0.3–0.5 mm (in vivo CT)	Density-based; ~10 HU under controlled acquisition	Hounsfield Units (HU), BMD, BV/TV, Tb.Th, Tb.Sp, volumetric descriptors
**Optical imaging**	~1–3 mm (fluorescence, depth-dependent)	~10^{-12} mol/L (fluorescence); substrate-dependent (bioluminescence)	Fluorescence intensity, fluorophore concentration, bioluminescence flux, FRET efficiency
**Photoacoustic**	~100–500 µm (depth-dependent)	µM range for haemoglobin-based metrics	Oxygen saturation (sO_2), total haemoglobin (HbT), chromophore distribution

Abbreviations: PET, Positron Emission Tomography; SPECT, Single Photon Emission Computed Tomography; MRI, Magnetic Resonance Imaging; CT, Computed Tomography; ADC, Apparent Diffusion Coefficient; DWI, Diffusion-Weighted Imaging; SUV, Standardized Uptake Value; AIF, Arterial Input Function; sO_2, oxygen saturation; HbT, total haemoglobin; EANM, European Association of Nuclear Medicine; ESMI, European Society for Molecular Imaging; ISMRM, International Society for Magnetic Resonance in Medicine; QA, quality assurance; OCT, Optical Coherence Tomography; MSOT, Multispectral Optoacoustic Tomography; BMD, bone mineral density; BV/TV, bone volume/total volume; Tb.Th, trabecular thickness; Tb.Sp, trabecular separation; HU, Hounsfield Unit; BPND, binding potential; DVR, distribution volume ratio; FRET, Förster resonance energy transfer; CEST, chemical exchange saturation transfer; TMS, tetramethylsilane; HFUS, high-frequency ultrasound; QSM, quantitative susceptibility mapping; MRS, magnetic resonance spectroscopy; IVIM, intravoxel incoherent motion; DKI, diffusion kurtosis imaging; CPMG, Carr-Purcell-Meiboom-Gill.

Part B. Modality scores with justification and key references - Supplementary Table S3

The following table reports the score assigned to each modality for each dimension, with a brief justification based on the evidence reviewed and the key references supporting each assessment. Where a modality appears across multiple rows, the modality name is shown only in the first row for readability.

Modality	Dimension	Score	Justification	Key references
**PET**	Absolute quantification	**5**	Traceable absolute quantification via SUV and kinetic parameters; validated in multicentre preclinical PET/CT studies with ex vivo biodistribution cross-validation.	(Chomet et al., 2021; Kuntner et al., 2024; McDougald et al., 2020)
**PET**	Technical variance controllability	**5**	Multicentre studies demonstrate inter-system variability is measurable and controllable through cross-platform calibration and structured QA programmes.	(Balber et al., 2025; Kuntner et al., 2025; Mannheim et al., 2019)
**PET**	Harmonization maturity	**5**	EANM/ESMI international guidelines formally adopted; multicentre standardization protocols published and validated; most mature harmonization ecosystem among reviewed modalities.	(Mannheim et al., 2019; McDougald et al., 2020; Vanhove et al., 2024)
**PET**	AI integration readiness	**4**	AI-assisted reconstruction and AIF estimation validated on preclinical data with demonstrated variance reduction; cross-platform prospective validation not yet consolidated.	(Mannheim et al., 2019; Reader & Pan, 2023)
**PET**	Cross-species applicability	**3**	Validated primarily in murine models; feasibility demonstrated in zebrafish and in ovo with pronounced scale-dependent challenges requiring formal metrological re-validation.	(Balber et al., 2025; Mannheim et al., 2025)
**PET**	Multiparametric depth	**2**	Primarily one quantitative parameter per acquisition (tracer concentration or kinetic constants); multi-tracer protocols require separate sessions and are not standardized.	(Raccagni et al., 2018; Vanhove et al., 2024)
**SPECT**	Absolute quantification	**3**	Absolute activity quantification achievable with CT-based attenuation correction and calibrated iterative reconstruction but more sensitive to calibration stability than PET.	(Bruzgo-Grzybko et al., 2025; Willowson & Bailey, 2024)
**SPECT**	Technical variance controllability	**3**	EANM/ESMI QA procedures available but less detailed than PET; main variance sources identified with partially standardized control strategies.	(Vanhove et al., 2024; Willowson & Bailey, 2024)
**SPECT**	Harmonization maturity	**3**	Included in EANM/ESMI framework but guidelines less detailed than PET; consensus recommendations available with partial adoption.	(Mannheim et al., 2018; Vanhove et al., 2024)
**SPECT**	AI integration readiness	**3**	Advanced computational reconstruction methods validated for specific SPECT applications; AI integration less developed than PET or MRI.	(Willowson & Bailey, 2024)
**SPECT**	Cross-species applicability	**3**	Applied in murine and other small animal models; cross-species framework less developed than PET; formal metrological re-validation across species not consolidated.	(Bruzgo-Grzybko et al., 2025; Mannheim et al., 2018)

Modality	Dimension	Score	Justification	Key references
**SPECT**	Multiparametric depth	**2**	Primarily radiotracer distribution parameters; multiparametric capability structurally limited by physics of the method similarly to PET.	(Bruzgo-Grzybko et al., 2025; Willowson & Bailey, 2024)
**MRI**	Absolute quantification	**3**	Absolute quantification achievable for specific parameters (e.g. ADC) with standardized protocols and phantoms; vendor-dependent offsets and protocol heterogeneity limit universal traceability.	(Jelescu et al., 2025; Malyarenko et al., 2023; Pickup et al., 2022)
**MRI**	Technical variance controllability	**3**	Phantom-based variance characterization available for DWI; between-session variability formally quantified for relaxometry; vendor heterogeneity remains an unresolved challenge.	(Jelescu et al., 2025; Malyarenko et al., 2023; Roudi et al., 2024)
**MRI**	Harmonization maturity	**3**	ISMRM consensus recommendations in development for preclinical DWI; absent for most other MRI techniques; no universally adopted phantom standards.	(Jelescu et al., 2025; Mannheim et al., 2018)]
**MRI**	AI integration readiness	**5**	AI fully integrated in MRI quantitative pipelines including MR Fingerprinting, AI-assisted reconstruction, deep learning segmentation, and parametric mapping across multiple validated applications.	(Duan et al., 2024; Zhu et al., 2025)
**MRI**	Cross-species applicability	**5**	Validated in mouse, rat, rabbit and other models; widest cross-species applicability among reviewed modalities, supported by flexible acquisition frameworks and absence of radiotracer constraints.	(Albrecht et al., 2019; Guo et al., 2022; Zhu et al., 2025)
**MRI**	Multiparametric depth	**5**	Simultaneous extraction of T1, T2, diffusion, perfusion, susceptibility, and metabolic parameters demonstrated in validated protocols; highest multiparametric depth among reviewed modalities.	(Herrmann et al., 2016; Roudi et al., 2024; Zhu et al., 2025)
**CT / micro-CT**	Absolute quantification	**5**	Hounsfield Unit-based metrics traceable to physical attenuation units; reproducibility of densitometric and volumetric descriptors formally validated with repeatability coefficients.	(Clark & Badea, 2021; Oliviero et al., 2022)
**CT / micro-CT**	Technical variance controllability	**4**	Validated control strategies for main variance sources (beam hardening, scatter, partial volume) across skeletal, pulmonary, and tumour applications; cross-platform accreditation less developed than PET.	(Brown et al., 2024; Christiansen, 2016; Oliviero et al., 2022)
**CT / micro-CT**	Harmonization maturity	**3**	Consensus recommendations available for specific applications; formal international accreditation framework absent; cross-platform reproducibility of radiomic features protocol-dependent.	(Brown et al., 2024; Clark & Badea, 2021)
**CT / micro-CT**	AI integration readiness	**5**	Fully automated deep learning pipelines prospectively validated for lung fibrosis densitometry; AI-assisted segmentation benchmarked against multi-annotator reference standards.	(Buccardi et al., 2023; Cheng et al., 2025; Jensen et al., 2024; Vincenzi et al., 2022)
**CT / micro-CT**	Cross-species applicability	**5**	Validated in mouse, rat, and other small animal models for skeletal, pulmonary, vascular, and tumour applications; flexible framework applicable across species without tracer constraints.	(Ferrini et al., 2025; Jensen et al., 2024; Pereira-Rosa et al., 2024)

Modality	Dimension	Score	Justification	Key references
**CT / micro-CT**	Multiparametric depth	**2**	Primarily attenuation-based structural and densitometric parameters; contrast-enhanced protocols extend to vascular metrics but within the same physical domain.	(Ashton et al., 2015; Clark & Badea, 2021)
**Optical imaging**	Absolute quantification	**1**	Absolute quantification intrinsically confounded by tissue scattering, depth-dependent attenuation, and subject-specific optical properties; no traceability to universal reference units.	(Deng et al., 2022; Kim & Lee, 2022; Thompson et al., 2023)
**Optical imaging**	Technical variance controllability	**1**	Technical variance sources (injection variability, instrument sensitivity, optical tissue properties) poorly characterized at systematic framework level; control strategies limited.	(Kim & Lee, 2022; Smith et al., 2023)
**Optical imaging**	Harmonization maturity	**1**	No international accreditation framework; standardization recommendations proposed by single groups but not internationally adopted.	(Kim & Lee, 2022; Mannheim et al., 2018)
**Optical imaging**	AI integration readiness	**5**	AI fully integrated in high-content optical screening, radiomics-based feature extraction, deep learning-assisted OCT, and automated multiplexed phenotyping across multiple validated preclinical applications.	(Bini et al., 2024; Li et al., 2023; Sturtzel et al., 2025)
**Optical imaging**	Cross-species applicability	**5**	Compatible with zebrafish, murine, and organoid models; widest biological scale coverage among reviewed modalities, enabled by optical transparency of zebrafish and flexibility of acquisition systems.	(Cani et al., 2026; Kugler et al., 2022; Turrini et al., 2023)
**Optical imaging**	Multiparametric depth	**4**	Multiple parameters extractable including morphometric, functional, and molecular readouts; particularly rich in zebrafish platforms; less systematically integrated than MRI.	(Lichtenegger et al., 2022; Mitovic et al., 2025; Smith et al., 2023)
**Photoacoustic**	Absolute quantification	**2**	Relative quantification of sO2 and HbT feasible with spectral unmixing; absolute quantification strongly dependent on depth-varying fluence correction and tissue acoustic heterogeneity.	(Humbert et al., 2020; Upputuri & Pramanik, 2016)
**Photoacoustic**	Technical variance controllability	**2**	Main variance sources (fluence attenuation, spectral colouring, acoustic heterogeneity) identifiable but control strategies still in development; separation of biological and instrumental variance partially achieved.	(Jelescu et al., 2025; Malyarenko et al., 2023; Pickup et al., 2022)
**Photoacoustic**	Harmonization maturity	**2**	No international accreditation framework; consensus protocols for quantitative validation emerging rather than established.	(Gargiulo et al., 2019; Upputuri & Pramanik, 2016)
**Photoacoustic**	AI integration readiness	**3**	Advanced computational spectral unmixing and eigenspectral decomposition validated for specific applications; cross-platform AI generalizability not established.	(Olefir et al., 2019)
**Photoacoustic**	Cross-species applicability	**3**	Applied in zebrafish and murine models; feasibility demonstrated but formal metrological re-validation across species not consolidated.	(Humbert et al., 2020; Omar et al., 2016)

Modality	Dimension	Score	Justification	Key references
**Photoacoustic**	Multiparametric depth	3	Multiple parameters extractable (sO2, HbT, chromophore distribution) in dual-modality configurations; integration less systematic than MRI.	(Sun et al., 2024)

Abbreviations: PET, Positron Emission Tomography; SPECT, Single Photon Emission Computed Tomography; MRI, Magnetic Resonance Imaging; CT, Computed Tomography; ADC, Apparent Diffusion Coefficient; DWI, Diffusion-Weighted Imaging; SUV, Standardized Uptake Value; AIF, Arterial Input Function; sO2, oxygen saturation; HbT, total haemoglobin; EANM, European Association of Nuclear Medicine; ESMI, European Society for Molecular Imaging; ISMRM, International Society for Magnetic Resonance in Medicine; QA, quality assurance; OCT, Optical Coherence Tomography; MSOT, Multispectral Optoacoustic Tomography.

References

Albrecht, J., Polenz, D., Kühl, A. A., Rogasch, J. M. M., Leder, A., Sauer, I. M., Babos, M., Mócsai, G., Beindorff, N., Steffen, I. G., Brenner, W., & Koziolek, E. J. (2019). Diffusion-weighted magnetic resonance imaging using a preclinical 1 T PET/MRI in healthy and tumor-bearing rats. *EJNMMI Research*, *9*(1), 21. https://doi.org/10.1186/s13550-019-0489-6

Ashton, J. R., West, J. L., & Badea, C. T. (2015). In vivo small animal micro-CT using nanoparticle contrast agents. *Frontiers in Pharmacology*, *6*. https://doi.org/10.3389/fphar.2015.00256

Balber, T., Benčurová, K., Mayrhofer, M., Friske, J., Haas, M., Kuntner, C., Helbich, T. H., Hacker, M., Mitterhauser, M., & Rausch, I. (2025). Quantitative accuracy of preclinical in ovo PET/MRI: influence of attenuation and quantification methods. *EJNMMI Physics*, *12*(1), 5. https://doi.org/10.1186/s40658-024-00714-3

Bini, F., Missori, E., Pucci, G., Pasini, G., Marinozzi, F., Forte, G. I., Russo, G., & Stefano, A. (2024). Preclinical Implementation of matRadiomics: A Case Study for Early Malformation Prediction in Zebrafish Model. *Journal of Imaging*, *10*(11), 290. https://doi.org/10.3390/jimaging10110290

Brown, K. H., Kerr, B. N., Pettigrew, M., Connor, K., Miller, I. S., Shiels, L., Connolly, C., McGarry, C. K., Byrne, A. T., & Butterworth, K. T. (2024). A comparative analysis of preclinical computed tomography radiomics using cone-beam and micro-computed tomography scanners. *Physics and Imaging in Radiation Oncology*, *31*, 100615. https://doi.org/10.1016/j.phro.2024.100615

Bruzgo-Grzybko, M., Kalita, I. S., Olichwier, A. J., Bielicka, N., Chabielska, E., & Gromotowicz-Poplawska, A. (2025). Preclinical PET and SPECT Imaging in Small Animals: Technologies, Challenges and Translational Impact. *Cells*, *15*(1), 73. https://doi.org/10.3390/cells15010073

Buccardi, M., Ferrini, E., Pennati, F., Vincenzi, E., Ledda, R. E., Grandi, A., Buseghin, D., Villetti, G., Sverzellati, N., Aliverti, A., & Stellari, F. F. (2023). A fully automated micro-CT deep learning approach for precision preclinical investigation of lung fibrosis progression and response to therapy. *Respiratory Research*, *24*(1), 126. https://doi.org/10.1186/s12931-023-02432-3

Cani, O., Zannotti, L., De Vita, A., Liverani, C., Vanni, S., & Miserocchi, G. (2026). Zebrafish tumor xenograft models for drug-screening: a systematic review of methods for treatment assessment. *Journal of Translational Medicine*. https://doi.org/10.1186/s12967-026-07787-x

Cheng, H., Gao, T., Sun, Y., Huang, F., Gu, X., Shan, C., Luo, S., & Wang, B. (2025). AI-assisted semiquantitative measurement of murine bleomycin-induced lung fibrosis using in vivo micro-CT: an end-to-end approach. *American Journal of Physiology-Cell Physiology*, *329*(2), C659–C674. https://doi.org/10.1152/ajpcell.00604.2024

Chomet, M., Schreurs, M., Vos, R., Verlaan, M., Kooijman, E. J., Poot, A. J., Boellaard, R., Windhorst, A. D., van Dongen, G. A., Vugts, D. J., Huisman, M. C., & Beaino, W. (2021). Performance of nanoScan PET/CT and PET/MR for quantitative imaging of 18F and 89Zr as compared with ex vivo biodistribution in tumor-bearing mice. *EJNMMI Research*, *11*(1), 57. https://doi.org/10.1186/s13550-021-00799-2

Christiansen, B. A. (2016). Effect of micro-computed tomography voxel size and segmentation method on trabecular bone microstructure measures in mice. *Bone Reports*, *5*, 136–140. https://doi.org/10.1016/j.bonr.2016.05.006

Clark, D. P., & Badea, C. T. (2021). Advances in micro-CT imaging of small animals. *Physica Medica*, *88*, 175–192. https://doi.org/10.1016/j.ejmp.2021.07.005

Deng, Z., Xu, X., Iordachita, I., Dehghani, H., Zhang, B., Wong, J. W., & Wang, K. K.-H. (2022). Mobile bioluminescence tomography-guided system for pre-clinical radiotherapy research. *Biomedical Optics Express*, *13*(9), 4970. https://doi.org/10.1364/BOE.460737

Duan, Z., Tao, J., Liu, W., Liu, Y., Fang, S., Yang, Y., Liu, X., Deng, X., Song, Y., & Wang, S. (2024). Correlation of IVIM/DKI Parameters with Hypoxia Biomarkers in Fibrosarcoma Murine Models: Direct Control of MRI and Pathological Sections. *Academic Radiology*, *31*(3), 1014–1023. https://doi.org/10.1016/j.acra.2023.08.021

Ferrini, E., Buccardi, M., & Stellari, F. F. (2025). *In Vivo Micro-CT Imaging for Quantitative Longitudinal Assessment of Pulmonary Diseases in Small Animals* (pp. 207–232). https://doi.org/10.1007/978-1-0716-4418-8_14

Gargiulo, S., Albanese, S., & Mancini, M. (2019). State-of-the-Art Preclinical Photoacoustic Imaging in Oncology: Recent Advances in Cancer Theranostics. *Contrast Media & Molecular Imaging*, *2019*, 1–24. https://doi.org/10.1155/2019/5080267

Guo, J., Sun, W., Dong, C., Wu, Z., Li, X., Zhou, R., & Xu, W. (2022). Intravoxel incoherent motion imaging combined with diffusion kurtosis imaging to assess the response to radiotherapy in a rabbit VX2 malignant bone tumor model. *Cancer Imaging*, *22*(1), 47. https://doi.org/10.1186/s40644-022-00488-w

Herrmann, K., Erokwu, B. O., Johansen, M. L., Basilion, J. P., Gulani, V., Griswold, M. A., Flask, C. A., & Brady-Kalnay, S. M. (2016). Dynamic Quantitative T1 Mapping in Orthotopic Brain Tumor Xenografts. *Translational Oncology*, *9*(2), 147–154. https://doi.org/10.1016/j.tranon.2016.02.004

Humbert, J., Will, O., Peñate-Medina, T., Peñate-Medina, O., Jansen, O., Both, M., & Glüer, C.-C. (2020). Comparison of photoacoustic and fluorescence tomography for the in vivo imaging of ICG-labelled liposomes in the medullary cavity in mice. *Photoacoustics*, *20*, 100210. https://doi.org/10.1016/j.pacs.2020.100210

Jelescu, I. O., Grussu, F., Ianus, A., Hansen, B., Barrett, R. L. C., Aggarwal, M., Michielse, S., Nasrallah, F., Syeda, W., Wang, N., Veraart, J., Roebroeck, A., Bagdasarian, A. F., Eichner, C., Sepehrband, F., Zimmermann, J., Soustelle, L., Bowman, C., Tendler, B. C., … Schilling, K. G. (2025). Considerations and recommendations from the <scp>ISMRM</scp> diffusion study group for preclinical diffusion <scp>MRI</scp> : Part 1: In vivo small-animal imaging. *Magnetic Resonance in Medicine*, *93*(6), 2507–2534. https://doi.org/10.1002/mrm.30429

Jensen, M., Clemmensen, A., Hansen, J. G., van Krimpen Mortensen, J., Christensen, E. N., Kjaer, A., & Ripa, R. S. (2024). 3D whole body preclinical micro-CT database of subcutaneous tumors in mice with annotations from 3 annotators. *Scientific Data*, *11*(1), 1021. https://doi.org/10.1038/s41597-024-03814-y

Kim, S. J., & Lee, H.-Y. (2022). In vivo molecular imaging in preclinical research. *Laboratory Animal Research*, *38*(1), 31. https://doi.org/10.1186/s42826-022-00142-3

Kugler, E. C., Frost, J., Silva, V., Plant, K., Chhabria, K., Chico, T. J. A., & Armitage, P. A. (2022). Zebrafish vascular quantification: a tool for quantification of three-dimensional zebrafish cerebrovascular architecture by automated image analysis. *Development*, *149*(3). https://doi.org/10.1242/dev.199720

Kuntner, C., Alcaide, C., Anestis, D., Bankstahl, J. P., Boutin, H., Brasse, D., Elvas, F., Forster, D., Rouchota, M. G., Tavares, A., Teuter, M., Wanek, T., Zachhuber, L., & Mannheim, J. G. (2024). Optimizing SUV Analysis: A Multicenter Study on Preclinical FDG-PET/CT Highlights the Impact of Standardization. *Molecular Imaging and Biology*, *26*(4), 668–679. https://doi.org/10.1007/s11307-024-01927-9

Kuntner, C., Friske, J., Stessl, A., Haas, M., Breyer, L. L., Wanek, T., Hacker, M., Helbich, T., & Rausch, I. (2025). Assessing PET performance, image quality, and attenuation correction in the presence of RF coils within a 9.4 T MRI for preclinical simultaneous imaging. *EJNMMI Physics*, *12*(1), 63. https://doi.org/10.1186/s40658-025-00771-2

Li, K., Liu, B., Wang, Z., Li, Y., Li, H., Wu, S., & Li, Z. (2023). Quantitative characterization of zebrafish development based on multiple classifications using Mueller matrix OCT. *Biomedical Optics Express*, *14*(6), 2889. https://doi.org/10.1364/BOE.488614

Lichtenegger, A., Tamaoki, J., Licandro, R., Mori, T., Mukherjee, P., Bian, L., Greutter, L., Makita, S., Wöhrer, A., Matsusaka, S., Kobayashi, M., Baumann, B., & Yasuno, Y. (2022). Longitudinal investigation of a xenograft tumor zebrafish model using polarization-sensitive optical coherence tomography. *Scientific Reports*, *12*(1), 15381. https://doi.org/10.1038/s41598-022-19483-z

Malyarenko, D., Amouzandeh, G., Pickup, S., Zhou, R., Manning, H. C., Gammon, S. T., Shoghi, K. I., Quirk, J. D., Sriram, R., Larson, P., Lewis, M. T., Pautler, R. G., Kinahan, P. E., Muzi, M., & Chenevert, T. L. (2023). Evaluation of Apparent Diffusion Coefficient Repeatability and Reproducibility for Preclinical MRIs Using Standardized Procedures and a Diffusion-Weighted Imaging Phantom. *Tomography*, *9*(1), 375–386. https://doi.org/10.3390/tomography9010030

Mannheim, J. G., Kara, F., Doorduin, J., Fuchs, K., Reischl, G., Liang, S., Verhoye, M., Gremse, F., Mezzanotte, L., & Huisman, M. C. (2018). Standardization of Small Animal Imaging—Current Status and Future Prospects. *Molecular Imaging and Biology*, *20*(5), 716–731. https://doi.org/10.1007/s11307-017-1126-2

Mannheim, J. G., Lan, W., Conti, M., Siedler, F., Krueger, M. A., Herfert, K., la Fougère, C., & Schmidt, F. P. (2025). Feasibility of in vivo small animal imaging using a clinical total-body PET/CT system. *EJNMMI Physics*, *12*(1), 71. https://doi.org/10.1186/s40658-025-00782-z

Mannheim, J. G., Mamach, M., Reder, S., Traxl, A., Mucha, N., Disselhorst, J. A., Mittelhäuser, M., Kuntner, C., Thackeray, J. T., Ziegler, S., Wanek, T., Bankstahl, J. P., & Pichler, B. J. (2019). Reproducibility and Comparability of Preclinical PET Imaging Data: A Multicenter Small-Animal PET Study. *Journal of Nuclear Medicine*, *60*(10), 1483–1491. https://doi.org/10.2967/jnumed.118.221994

McDougald, W., Vanhove, C., Lehnert, A., Lewellen, B., Wright, J., Mingarelli, M., Corral, C. A., Schneider, J. E., Plein, S., Newby, D. E., Welch, A., Miyaoka, R., Vandenberghe, S., & Tavares, A. A. S. (2020). Standardization of Preclinical PET/CT Imaging to Improve Quantitative Accuracy, Precision, and Reproducibility: A Multicenter Study. *Journal of Nuclear Medicine*, *61*(3), 461–468. https://doi.org/10.2967/jnumed.119.231308

Mitovic, N., Kovacevic, S., Nesovic Ostojic, J., Puflovic, D., & S. Stankovic, M. (2025). Functional Cardiac Imaging in Zebrafish Embryos Using Standard Microscopy and Video Analysis: Applications in Environmental and Biomedical Research. *Journal of Visualized Experiments*, (224). https://doi.org/10.3791/68941

Olefir, I., Ghazaryan, A., Yang, H., Malekzadeh-Najafabadi, J., Glasl, S., Symvoulidis, P., O'Leary, V. B., Sergiadis, G., Ntziachristos, V., & Ovsepian, S. V. (2019). Spatial and Spectral Mapping and Decomposition of Neural Dynamics and Organization of the Mouse Brain with Multispectral Optoacoustic Tomography. *Cell Reports*, *26*(10), 2833-2846.e3. https://doi.org/10.1016/j.celrep.2019.02.020

Oliviero, S., Cheong, V. S., Roberts, B. C., Orozco Diaz, C. A., Griffiths, W., Bellantuono, I., & Dall'Ara, E. (2022). Reproducibility of Densitometric and Biomechanical Assessment of the Mouse Tibia From In Vivo Micro-CT Images. *Frontiers in Endocrinology*, *13*. https://doi.org/10.3389/fendo.2022.915938

Omar, M., Rebling, J., Wicker, K., Schmitt-Manderbach, T., Schwarz, M., Gateau, J., López-Schier, H., Mappes, T., & Ntziachristos, V. (2016). Optical imaging of post-embryonic zebrafish using multi orientation raster scan optoacoustic mesoscopy. *Light: Science & Applications*, *6*(1), e16186–e16186. https://doi.org/10.1038/lsa.2016.186

Pereira-Rosa, A., Oliveira, T. S., Ferreira, M. S., Vianna-Barbosa, R. J., Wilmart-Gonçalves, T. C., Ortiga, T. M., & Bloise, F. F. (2024). Non-invasive Skeletal Muscle Quantification in Small Animals Using Micro-computed Tomography. *Journal of Visualized Experiments*, (213). https://doi.org/10.3791/67393

Pickup, S., Romanello, M., Gupta, M., Song, H., & Zhou, R. (2022). Dynamic Contrast-Enhanced MRI in the Abdomen of Mice with High Temporal and Spatial Resolution Using Stack-of-Stars Sampling and KWIC Reconstruction. *Tomography*, *8*(5), 2113–2128. https://doi.org/10.3390/tomography8050178

Raccagni, I., Belloli, S., Valtorta, S., Stefano, A., Presotto, L., Pascali, C., Bogni, A., Tortoreto, M., Zaffaroni, N., Daidone, M. G., Russo, G., Bombardieri, E., & Moresco, R. M. (2018). [18F]FDG and [18F]FLT PET for the evaluation of response to neo-adjuvant chemotherapy in a model of triple negative breast cancer. *PLOS ONE*, *13*(5), e0197754. https://doi.org/10.1371/journal.pone.0197754

Reader, A. J., & Pan, B. (2023). AI for PET image reconstruction. *The British Journal of Radiology*, *96*(1150). https://doi.org/10.1259/bjr.20230292

Roudi, R., Pisani, L. J., Pisani, F., Liang, T., & Daldrup-Link, H. E. (2024). Reproducibility and repeatability of quantitative T2 and T2* mapping of osteosarcomas in a mouse model. *European Radiology Experimental*, *8*(1), 74. https://doi.org/10.1186/s41747-024-00467-9

Smith, J. T., Sinsuebphon, N., Rudkouskaya, A., Michalet, X., Intes, X., & Barroso, M. (2023). In vivo quantitative FRET small animal imaging: Intensity versus lifetime-based FRET. *Biophysical Reports*, *3*(2), 100110. https://doi.org/10.1016/j.bpr.2023.100110

Sturtzel, C., Grissenberger, S., Wenninger-Weinzierl, A., & Distel, M. (2025). *High-Content Imaging-Based Screening for Anticancer Compounds in Zebrafish Xenografts* (pp. 153–162). https://doi.org/10.1007/978-1-0716-4418-8_9

Sun, Y., Wang, Y., Li, W., & Li, C. (2024). Real-time dual-modal photoacoustic and fluorescence small animal imaging. *Photoacoustics*, *36*, 100593. https://doi.org/10.1016/j.pacs.2024.100593

Thompson, W. R., Brecht, H.-P. F., Ivanov, V., Yu, A. M., Dumani, D. S., Lawrence, D. J., Emelianov, S. Y., & Ermilov, S. A. (2023). Characterizing a photoacoustic and fluorescence imaging platform for preclinical murine longitudinal studies. *Journal of Biomedical Optics*, *28*(03). https://doi.org/10.1117/1.JBO.28.3.036001

Turrini, L., Roschi, L., de Vito, G., Pavone, F. S., & Vanzi, F. (2023). Imaging Approaches to Investigate Pathophysiological Mechanisms of Brain Disease in Zebrafish. *International Journal of Molecular Sciences*, *24*(12), 9833. https://doi.org/10.3390/ijms24129833

Upputuri, P. K., & Pramanik, M. (2016). Recent advances toward preclinical and clinical translation of photoacoustic tomography: a review. *Journal of Biomedical Optics*, *22*(4), 041006. https://doi.org/10.1117/1.JBO.22.4.041006

Vanhove, C., Koole, M., Fragoso Costa, P., Schottelius, M., Mannheim, J., Kuntner, C., Warnock, G., McDougald, W., Tavares, A., & Bernsen, M. (2024). Preclinical SPECT and PET: Joint EANM and ESMI procedure guideline for implementing an efficient quality control programme. *European Journal of Nuclear Medicine and Molecular Imaging*, *51*(13), 3822–3839. https://doi.org/10.1007/s00259-024-06824-5

Vincenzi, E., Fantazzini, A., Basso, C., Barla, A., Odone, F., Leo, L., Mecozzi, L., Mambrini, M., Ferrini, E., Sverzellati, N., & Stellari, F. F. (2022). A fully automated deep learning pipeline for micro-CT-imaging-based densitometry of lung fibrosis murine models. *Respiratory Research*, *23*(1), 308. https://doi.org/10.1186/s12931-022-02236-x

Willowson, K. P., & Bailey, D. L. (2024). Evolving SPECT-CT technology. *British Journal of Radiology*. https://doi.org/10.1093/bjr/tqae200

Zhu, Y., Wang, G., Gu, Y., Zhao, W., Lu, J., Zhu, J., MacAskill, C. J., Dupuis, A., Griswold, M. A., Ma, D., Flask, C. A., & Yu, X. (2025). <scp>3D MR</scp> fingerprinting for dynamic contrast-enhanced imaging of whole mouse brain. *Magnetic Resonance in Medicine*, *93*(1), 67–79. https://doi.org/10.1002/mrm.30253
